# Characterization of chemotaxis in soybean symbiont *Bradyrhizobium diazoefficiens*

**DOI:** 10.1128/aem.00928-26

**Published:** 2026-06-24

**Authors:** Matthew B. Lubin, Daniel H. Teixeira, Brittany J. Belin

**Affiliations:** 1 Department of Embryology, Carnegie Science 88957, Baltimore, Maryland, USA; 2 Department of Biology, Johns Hopkins University 228291, Baltimore, Maryland, USA; The University of Tennessee Knoxville, Knoxville, Tennessee, USA

**Keywords:** *Bradyrhizobium*, soybean, chemotaxis, rhizobia

## Abstract

Symbiotic relationships between nitrogen-fixing soil bacteria and legumes provide nearly half of all biologically fixed nitrogen on Earth, playing a crucial role in sustainable agriculture. These relationships rely on bacterial navigation of complex, dynamic soil environments to reach their plant hosts. Central to this behavior are bacterial motility and chemotaxis, the ability to sense and move toward host-derived signals in the rhizosphere. In the soybean symbiont *Bradyrhizobium diazoefficiens* USDA110, motility is controlled by dual flagellar systems, and this strain contains three putative but uncharacterized chemotaxis operons (che1, che2, and che3). Using targeted deletions of all three predicted *cheA* genes, we show that che2 is the primary driver of chemotaxis toward soybean seed exudate in soft agar assays, and that the greater contribution of che2 vs. che1 to soft agar chemotaxis is not due to differences in CheA protein sequence. Interestingly, we also find that *B. diazoefficiens* mutants incapable of chemotaxis in semisolid media retain wild-type-like swimming speeds in aqueous media. These findings provide insight into how *B. diazoefficiens* coordinates its chemosensory systems to respond to its host plant.

**IMPORTANCE:**

Chemotaxis is crucial for the establishment of beneficial plant-microbe associations, yet mechanistic studies of chemotaxis have been limited to a handful of bacterial models. The soybean symbiont *Bradyrhizobium diazoefficiens* USDA110 is a commonly used soybean inoculant with exceptional nitrogen fixation efficiency, but the genetic control of chemotaxis in *B. diazoefficiens* has not been examined. Establishing *B. diazoefficiens* as a model of chemotaxis provides an opportunity to understand how multiple chemotaxis systems coordinate root colonization in this major agricultural symbiont and can enable comparative analyses of plant-microbe recognition strategies across agricultural bacteria.

## INTRODUCTION

Nitrogen-fixing bacteria are key players in the nitrogen cycle and convert atmospheric nitrogen into ammonia, thereby providing the building blocks for protein synthesis in all domains of life. In soil, symbiotic nitrogen-fixing strains known as rhizobia can provide ammonia directly to legume plant hosts (1). This symbiosis reduces the need for synthetic fertilizers and increases legume carbon sequestration, making rhizobia indispensable partners in sustainable agriculture. Given the high economic and environmental benefits of maintaining robust populations of rhizobia, it is important to understand how these microorganisms survive and navigate the soil habitat to establish beneficial associations with plants.

Natural soils are highly heterogeneous and dynamic environments that are relatively poor in nutrients, and soil-associated bacteria have evolved sophisticated motility and chemotaxis systems to sense and move toward nutrient gradients (2–4). Compared to bacteria in marine and animal environments, soil bacteria typically encode much higher copy numbers of chemotaxis receptors and signaling genes (5), suggesting that integrating multiple parallel chemosensory systems increases fitness in the soil niche (6–9). The relative contributions of individual chemotaxis (*che*) gene orthologs and operons have been examined in three model rhizobia: *Rhizobium leguminosarum*, *Sinorhizobium meliloti*, and *Azospirillum brasilense* (8, 10, 11). These studies revealed that the molecular functions of *che* genes and operons can be organism-specific, and they do not always contribute directly, or exclusively, to chemotaxis. For example, *che* genes can regulate pathways (12), such as biofilm formation, exopolysaccharide production (13), nitrogen metabolism (14, 15), and cell division (16), as well as chemotaxis-independent aspects of root colonization and nodulation (15, 17–22). Thus, it is difficult to predict whether individual *che* gene operons regulate chemotaxis *a priori*.

Soybean is the most globally dominant legume crop by production volume, and it forms efficient symbiosis with the rhizobium *Bradyrhizobium diazoefficiens* USDA110 (8, 23, 24). This strain possesses competitive nodulation and is highly motile (24–29). Its motility is primarily driven by its two flagellar systems: a subpolar flagellum that drives swimming through water-filled pores, and multiple lateral flagella that facilitate movement across surfaces and through viscous environments (30, 31). Although regulation of flagellar genes in *B. diazoefficiens* has been shown to interact with metabolic and environmental sensing pathways (32, 33), and this strain can move directionally toward soybean exudate compounds (34–36), the genes required for *B. diazoefficiens* chemotaxis are unknown.

Canonical bacterial chemotaxis requires a signal transduction pathway involving methyl-accepting chemotaxis proteins (MCPs), the histidine kinase CheA, and the response regulator CheY (37–39). Analysis of the *B. diazoefficiens* USDA110 genome revealed 36 MCPs and three chemotaxis operons, each containing its own *cheA* gene (8, 40, 41). Here, we use gene deletions to analyze the role of each *cheA* ortholog in chemotactic signaling and motility control. This study establishes *B. diazoefficiens* as a new model for chemotaxis in soil bacteria, which may provide a foundation for engineering improved *Bradyrhizobium* spp. inoculants in agriculture (2, 42–44).

## MATERIALS AND METHODS

### Media recipes

Three media formulations were used in this study: rich AG medium, minimal AG medium, and motility medium. All of these share a common mineral base and differ only in their carbon content. The shared base consists of 0.88 mM Na₂HPO₄, 1.8 mM Na₂SO₄, 6 mM NH₄Cl, 0.73 mM MgSO₄ (anhydrous), 30 μM FeCl₃, 90 μM CaCl₂·2H₂O, 5.5 mM HEPES, and 5.6 mM MES monohydrate, adjusted to pH 6.6 prior to autoclaving. Rich AG medium was additionally supplemented with 1 g/L L-arabinose, 1 g/L sodium gluconate, and 1 g/L yeast extract (Bacto, BD Biosciences); minimal AG medium contained 0.5 g/L L-arabinose and 0.5 g/L sodium gluconate without yeast extract; and motility medium contained 0.1 g/L L-arabinose and 0.1 g/L sodium gluconate without yeast extract. Solid media were prepared by addition of agar (Bacto, BD Biosciences) to a final concentration of 1.5% (wt/vol) for plating or 0.3% (wt/vol) for semisolid chemotaxis assays.

### Bacterial strains and cultivation

Wild-type strains in this study refer to *Bradyrhizobium diazoefficiens* USDA110 *spc*4 (https://doi.org/10.13145/bacdive133983.20251217.10), which was used as the parent strain for all *che* gene genetic modifications. The non-motile, flagella-deficient strain Δ*fla* (LP 6543) was provided by A. R. Lodeiro (Universidad Nacional de La Plata, Argentina) and was described previously (27, 30). A complete list of all strains used in this study, including genotypes and sources, is provided in Table S1. For all strains, glycerol stocks were streaked onto agar plates containing rich AG medium and incubated at 30°C for 3–5 days. Starter cultures (5 mL rich AG) were inoculated from single colonies on plates and grown to mid-exponential phase (OD₆₀₀ = 0.4–0.7) at 30°C with shaking at 250 rpm prior to each experiment.

### Generation of deletion strains

We generated deletion mutants of *cheA1* (*bll0393*), *cheA2* (*blr2192*), *cheA3* (*blr2343*) (Table S1), and combinations thereof, using homologous recombination (45). For each deletion, we designed DNA constructs containing 1 kb sequences homologous to the regions upstream and downstream of the target gene. These sequences were synthesized by Twist Bioscience and cloned into the suicide vector pK18mobsacB, which carries kanamycin resistance and sucrose sensitivity markers. Full vector sequences can be found in Table S4.

Deletion plasmids were introduced into *Escherichia coli* S17 donor cells (ATCC 47055) by electroporation using a Bio-Rad Gene Pulser (2.5 kV, 200 Ω, 25 μF) and delivered to *B. diazoefficiens* USDA110 by conjugation. Conjugation mixtures were plated on rich AG medium plates containing carbenicillin (150 μg/mL) and kanamycin (200 μg/mL). Kanamycin-resistant colonies were then grown in non-selective rich AG medium and plated on rich AG/agar plates containing 5% sucrose for counterselection. Double and triple deletion mutants were generated by sequential rounds of homologous recombination and counterselection. Candidate deletion mutants were confirmed by Sanger sequencing (Azenta Bioscience).

### Soybean seed exudate preparation

Soybean seeds (Deer Creek, Hutchison) were surface-sterilized by rinsing three times with 90% ethanol, soaking for 5 min in a 5% bleach solution with mild agitation, and rinsing five times with nanopure water. Soybean seed exudate (sSE) was prepared by soaking 100 g of surface-sterilized soybean seeds in 100 mL of double-distilled water for 24 h at room temperature without agitation. The resulting aqueous solution was separated from the seeds and lyophilized at −80°C. The dried material (approx. 4 g) was then reconstituted in 10 mL of nanopure water to yield a “10×” concentrated solution of approximately 400 mg/mL. This 10× solution was sterilized by passage through a 0.2 μm membrane filter, flash-frozen with liquid nitrogen, and stored at −80°C for up to 6 months.

### Reverse transcription quantitative PCR (RT-qPCR)

Starter cultures grown to exponential phase were diluted to OD_600_ = 0.02 in rich AG medium and grown overnight to exponential phase. For each treatment, a 5 mL culture sample was transferred into a 15 mL conical tube, washed, and resuspended in 5 mL of motility medium with 500 μL of 10× sSE or nanopure water, and returned to the incubator for 8 h. After incubation, total RNA was extracted using the RNeasy Mini Kit (Qiagen) according to the manufacturer’s instructions, including initial lysozyme step and on-column DNase I digestion. First-strand cDNA synthesis was performed using 500 ng total RNA (quantified by NanoDrop, Thermo Scientific) with AMV reverse transcriptase (Promega) and random primers in 20 μL reactions following the manufacturer’s recommended protocol. RT-qPCR was conducted using Power SYBR Green Master Mix (Applied Biosystems). Each 20 μL reaction contained 10 μL 2× SYBR Green Master Mix, 0.2 μL each of 10 μM forward and reverse primers, 1 μL diluted cDNA (1:20), and 8.6 μL nuclease-free water. Thermal cycling was set according to the manufacturer’s instructions using a CFX Opus 96 Real-Time PCR System (Bio-Rad). Primer sequences can be found in Table S3.

### Growth curves

Starter cultures grown to exponential phase in rich AG were diluted to OD_600_ = 0.02 in minimal AG media. The media were supplemented with a 1:10 dilution of 10× sSE or an equivalent volume of distilled water. Two hundred microliter aliquots of each bacterial genotype were transferred to individual wells of a 96-well plate (Corning) in five wells per strain and condition. Wells were overlaid with 30 μL of mineral oil to prevent evaporation.

Growth was monitored using a Biotek Epoch 2 microplate reader maintained at 30°C with continuous orbital shaking. OD_600_ measurements were recorded every 2 h for 96 h. Between readings, the plate was shaken at 180 rpm with an orbital amplitude of 2 mm to ensure adequate aeration. Growth curves were generated by plotting the mean OD_600_ values against time, with shading representing the standard deviation of three biological replicates.

### Semisolid agar colony expansion assays

Motility medium with 0.3% agar was autoclaved, and 50 mL aliquots were poured into 12 cm square plates (Greiner Bio-One). Starter cultures grown to mid-exponential phase were back-diluted to OD_600_ = 0.02 in motility medium and grown again to early exponential phase (OD_600_ = 0.15–0.20). A 10 μL culture aliquot was added to the center of each quadrant of the plate.

Plates were incubated at 30°C in a humidified chamber. Inoculant expansion was documented by capturing images every 24 h for 7 days using a Panasonic Lumix DSLR camera mounted on a fixed stand. Colony diameters were quantified using ImageJ and compared between genotypes using one-way ANOVA between WT and each mutant strain at 7 days post-inoculation (dpi); *P*-values were computed in Python using scipy.stats on per-plate replicate values from a single experiment.

### Semisolid agar chemotaxis assays

Motility medium with 0.3% agar was autoclaved, and 50 mL aliquots were poured into 12 cm square plates (Greiner Bio-One). Starter cultures grown to mid-exponential phase were back-diluted to OD_600_ = 0.02 in motility medium and grown again to early exponential phase (OD_600_ = 0.15–0.20). A 10 μL culture aliquot was added to the center of each plate. Two sterile Whatman filter paper discs (6 mm diameter) were placed 4 cm away from the inoculation site on either side. One disc was saturated with 50 μL of concentrated soybean seed exudate, whereas the control disc on the opposite side received 50 μL of sterile water.

Plates were incubated at 30°C in a humidified chamber. Inoculant expansion was documented by capturing images every 24 h for 7 days using a Panasonic Lumix DSLR camera mounted on a fixed stand. Expansion was quantified using ImageJ, and the bias of the expansion toward sSE was calculated as the expansion distance from the center inoculation site toward sSE as a fraction of the expansion distance in both directions:


$$\frac{\text{expansion towards}\;\text{sSE}\;\text{(cm)}}{\text{expansion towards}\;\text{sSE}\;\text{(cm) + expansion towards control (cm)}\;}$$

Directional bias was compared between genotypes using one-way ANOVA between WT and each mutant strain at 7 days post-inoculation (dpi); *P*-values were computed in Python using scipy.stats on per-plate replicate values from a single experiment.

### Single cell tracking

*B. diazoefficiens* cultures were grown to early exponential phase (OD_600_ = 0.2) in rich AG medium. The membrane-permeable nucleic acid dye SYTO9 (Thermo Fisher Scientific) was added to 1 mL of culture to a 2.5 μM final concentration (1:2,000 dilution from a 5 mM stock) and incubated with shaking for 10 min at 30°C. Immediately following staining, bacteria were spun down in a tabletop centrifuge at 750 × *g* for 8 min, gently resuspended in sterile saline (0.9% NaCl), and diluted to OD_600_ = 0.05.

To track cells swimming in homogeneous media, a 10 μL aliquot of the bacterial resuspension was placed onto a glass slide fitted with a SecureSeal Imaging Spacer (Grace Biolabs, Product no. 654002) and sealed with a #1.5H coverslip. To track cells swimming under a nutrient gradient, cells were added to the center trough of a μ-Chemotaxis slide (ibidi) with either minimal AG medium on both sides (control) or with the right chamber filled with medium supplemented with 10× sSE, AG rich medium, or 10 mM glutamate, following the protocol described in reference 46. All channel ports were plugged before microscopy observation.

Swimming cells were visualized using a Zeiss LSM 980 inverted confocal microscope equipped with a 40× objective (NA 1.4) and kept in a temperature-controlled PeCon chamber at 30°C for the duration of imaging. Images were acquired in Airyscan Fast mode using 488 nm laser excitation and a 527–735 nm emission window, with the pinhole set to ~8 Airy units. Fluorescence was detected using a 32-element GaAsP-PMT detector at a frame rate of 17 frames per second with an Axiocam 712 CMOS camera. For each strain, multiple 30-second videos were recorded of different fields of view, capturing approximately 500 cell trajectories per condition.

Individual cell trajectories were determined from imaging data using a MATLAB script (Mathworks, MATLAB R2022b) adapted from reference 47. Trajectories from non-motile cells (*i.e.*, cells moving by Brownian motion only) were removed from the data set using a cell displacement cutoff filter that removed tracks for which no five-frame window contained a cumulative frame-to-frame displacement of at least 50 pixels (≈4.25 µm). This threshold was empirically validated by comparison to formaldehyde-killed control cells imaged on the same microscope (Fig. S3A; Table S3). For motile cell trajectories, “run” and “tumble” events were identified using a sliding three-frame window approach. A frame was classified as part of a run if the interior angle between the velocity vectors of the preceding and following frames was less than 60°, whereas a frame was classified as part of a tumble if the angle exceeded 60°. Consecutive frames of the same classification then were merged into single events. For run events, run durations were calculated as the number of consecutive run frames divided by the imaging frame rate, and run speeds were calculated as the mean of the instantaneous cell speed for every frame within the run.

For each replicate and strain, summary statistics (mean track speed, mean run speed, mean run length, mean run duration, run-to-non-run ratio) were computed as the mean of per-track values, and per-replicate means were compared across genotypes within each experiment by one-way ANOVA followed by Tukey’s HSD with α = 0.05. Across-experiment significance was assessed using one-way ANOVA on the six per-replicate means.

### Flow cytometry

Bacterial viability was assessed using the LIVE/DEAD BacLight Bacterial Viability Kit (Thermo Fisher Scientific, kit L7012). Cells grown in minimal AG medium to OD₆₀₀ = 0.5 were pelleted at 10,000 × *g* for 10 min, washed twice in motility medium, and diluted 1:10 in motility medium prior to staining. A heat-killed, dead-cell reference was prepared in parallel by incubating an aliquot of cells at 95°C for 10 min prior to mixing. Samples were stained in equal volumes of Component A (SYTO 9) and Component B (propidium iodide, PI) from kit L7012 in accordance with the manufacturer’s recommended protocol.

SYTO9 and PI fluorescence were determined using an Attune NxT Acoustic Focusing Cytometer running Attune NxT Software v3.2.1526.0. Forward scatter was collected at a photomultiplier voltage of 350 V. SYTO 9 fluorescence was collected through a 500–560 nm bandpass emission filter after 488 nm laser excitation, and PI fluorescence was collected through a 585/16 nm bandpass emission filter after 561 nm laser excitation. For each sample, 100,000 events were recorded. The percentage of live cells was calculated for each strain as (number of SYTO 9^+^ PI⁻ events / total gated events) × 100.

## RESULTS

### Organization and expression of chemotaxis genes in *B. diazoefficiens* USDA 110

**Fig 1:**
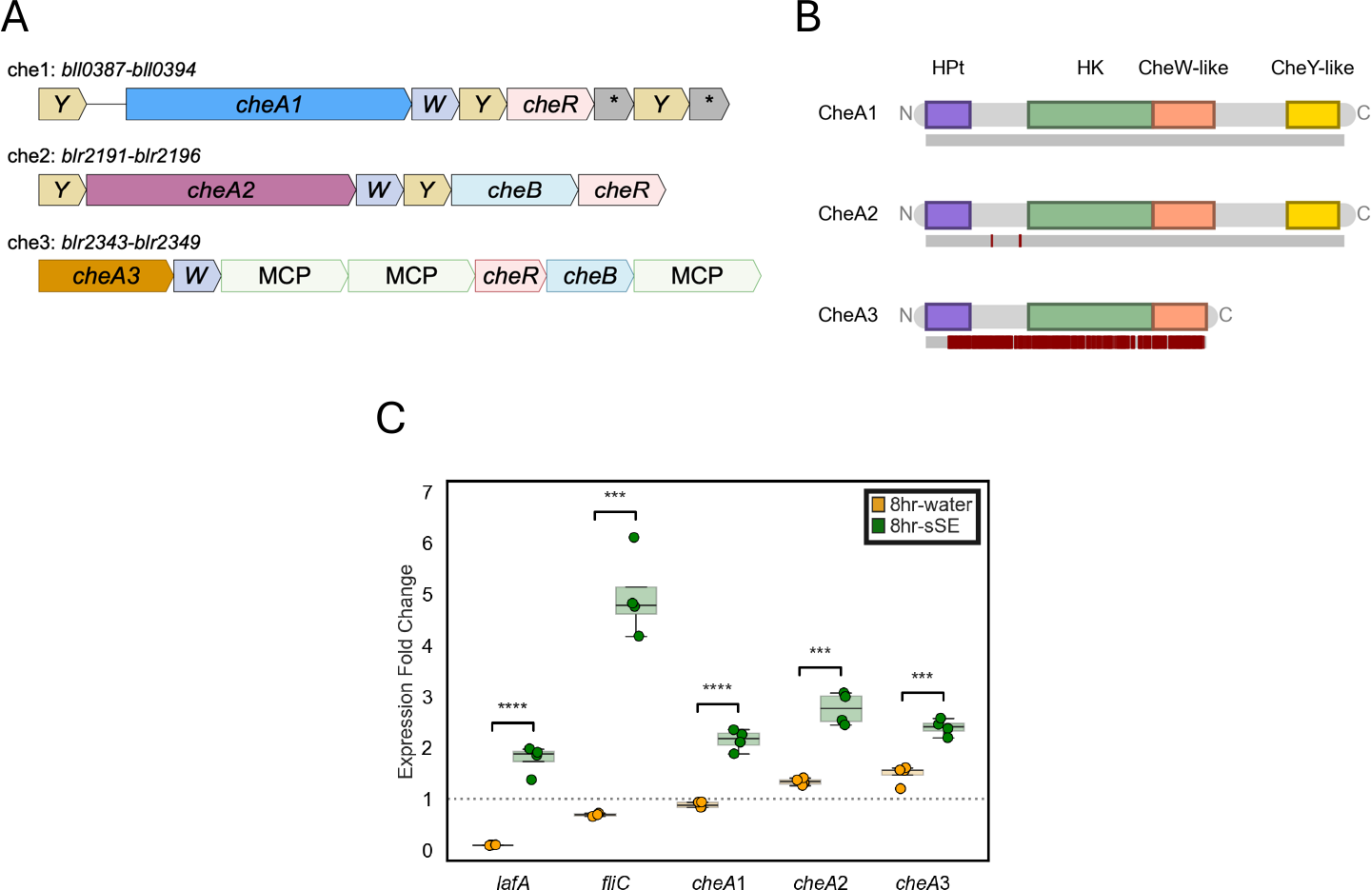
Chemotaxis genes of *B. diazoefficiens* USDA 110 are induced by soybean seed exudate. (**A**) Gene architecture of three predicted chemotaxis operons, each with characteristic *cheA* genes named according to their operon number. Asterisks (*) denote uncharacterized ORFs. (**B**) Predicted protein domain architecture of CheA1, CheA2, and CheA3, drawn to scale by amino acid length (CheA1 and CheA2 = 949 aa; CheA3 = 636 aa). Predicted domains include the histidine-phosphotransfer domain (HPt, purple), histidine-kinase catalytic domain (HK, green), CheW-like coupling domain (orange), and CheY-binding/CheY-like P2 domain (yellow). Vertical dark-red tick marks below each protein indicate amino acid positions where the sequence differs from CheA1. (**C**) Expression fold changes of the lateral-flagellum flagellin gene *lafA* (*blr3700*), the subpolar-flagellum flagellin gene *fliC* (*bll6855*), and the three *cheA* genes (*cheA1* = *bll0393*, *cheA2* = *blr2192*, *cheA3* = *blr2343*), measured by RT-qPCR after 8 h of incubation in motility medium with soybean seed exudate (sSE, green) or an equal volume of water (control, orange). Expression levels are reported relative to housekeeping gene *sigA* (*bll7349*). Statistical significance was determined by Welch’s two-sample *t*-test (8 h sSE vs 8 h water) for each gene: * *= P* < 0.05, ** = P < 0.01, *** = *P* < 0.001, **** = *P* < 0.0001.

The *B. diazoefficiens* USDA 110 genome has three predicted chemotaxis operons: che1, che2, and che3. Each operon contains an ortholog of the *cheA* histidine kinase gene; che1 and che2 also encode multiple *cheY* response regulator genes, whereas che3 does not encode a *cheY* ortholog (Fig. 1A). Sequence analysis of each predicted CheA protein (CheA1, CheA2, and CheA3) indicates that CheA1 and CheA2 both possess the canonical domain architecture required for phosphorelay signaling and share 99% overall amino acid sequence similarity (Fig. 1B). CheA3 shares only 19% pairwise identity with CheA1 and CheA2 and lacks the characteristic CheY-binding domain required for downstream Che protein activation (Fig. 1B).

Prior studies have shown that genes involved in chemotaxis are strongly induced by the presence of chemoattractants (13–15). We therefore tested whether each *cheA* gene could be induced by seed exudate from soybean (sSE), the native host of *B. diazoefficiens*. In liquid culture experiments, we used qRT-PCR and found that incubation of cells with sSE for 8 h induced significant upregulation of the lateral and subpolar flagellin genes *lafA* and *fliC*, as well as all three *cheA* genes (Fig. 1C).

### The *cheA2* gene is required for optimal chemotaxis in semisolid agar

**Fig 2:**
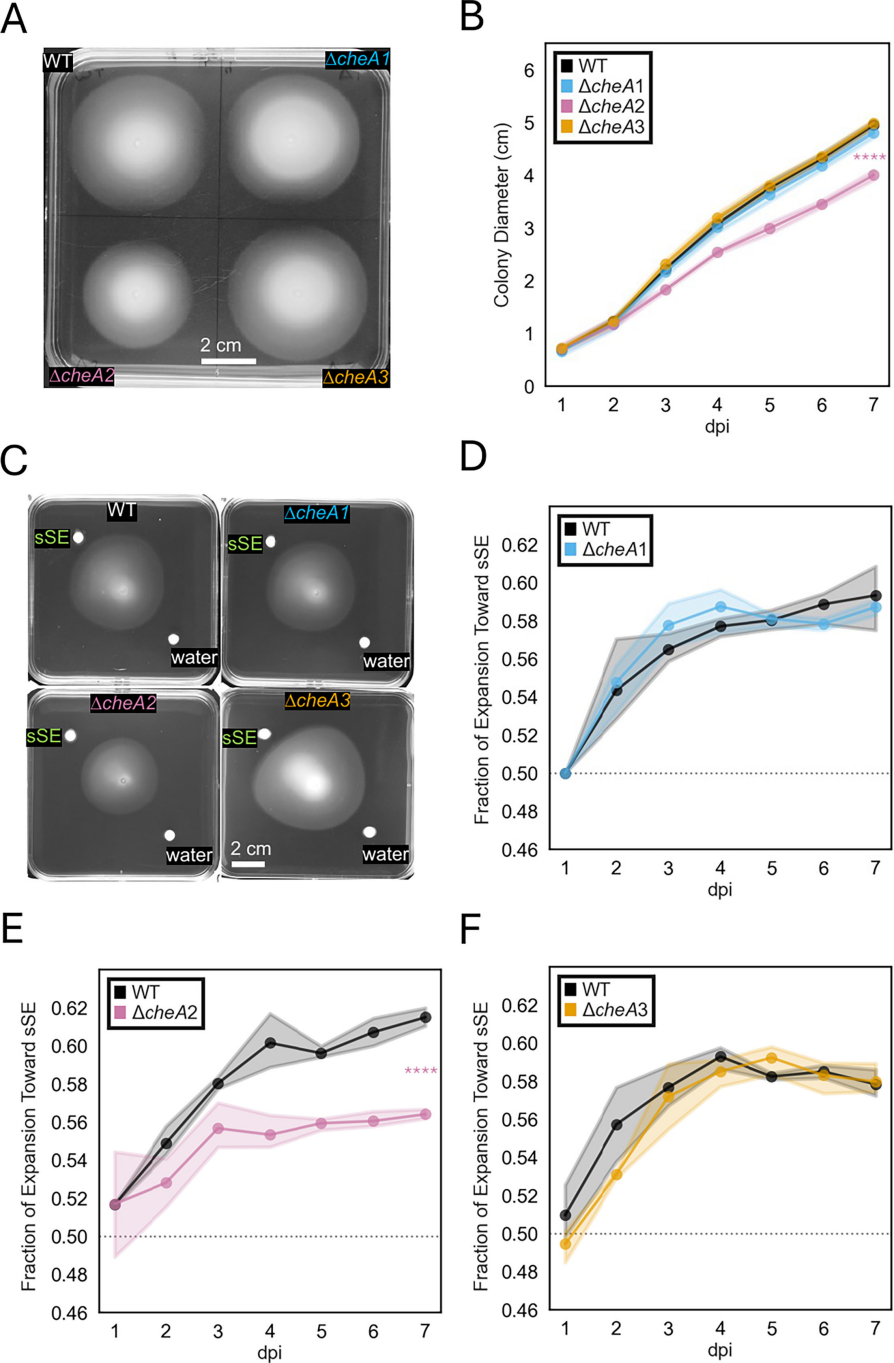
Loss of *cheA2* inhibits colony expansion and chemotaxis in semisolid media. (**A**) Representative images of expansion halos formed by WT and deletion mutants Δ*cheA1*, Δ*cheA2*, and Δ*cheA3* on semisolid (0.3% agar) minimal AG medium at 7 days post-inoculation (dpi). (**B**) Quantification of expansion-halo diameters for the strains shown in panel A over 7 days. Points are mean diameters across *N* = 3 plates per strain; shaded bands indicate the range of observed values. (**C**) Representative images of expansion of WT, Δ*cheA1*, Δ*cheA2*, and Δ*cheA3* on semisolid minimal AG plates containing two filter discs placed 4 cm from the inoculation point: one (left) saturated with 50 μL of 10 × concentrated sSE and the other (right) saturated with 50 μL of sterile water. Plates are shown at 7 dpi. (**D–F**) Fraction of colony expansion directed toward sSE over 7 days for (**D**) Δ*cheA1*, (**E**) Δ*cheA2*, and (**F**) Δ*cheA3*, each compared to WT measured on the same plate set. Points are means; shaded bands indicate the range of observed values; the dashed horizontal line at 0.5 represents no directional preference. Statistical significance was assessed by one-way ANOVA at 7 dpi for each mutant against WT: **** = *P* < 0.0001; comparisons without an asterisk are not significantly different (*P* > 0.05).

To investigate the functional roles of each che operon, we generated single *cheA* gene deletion mutants (Δ*cheA1*, Δ*cheA2*, Δ*cheA3*) via homologous recombination. Because deleting a *cheA* gene typically disrupts other downstream *che* genes within the same operon, the phenotypes of these mutants likely reflect the functions of their operons as a whole. We then examined these strains’ colony expansion rates in semisolid (0.3%) agar plates containing minimal AG medium, a condition where expansion depends on a combination of growth, swimming, and chemotactic navigation. Wild-type *B. diazoefficiens* formed characteristic expansion “halos” of swimming cells that grew radially from the inoculation site, reaching approximately 5 cm in diameter after 7 days (Fig. 2A). The deletion mutants Δ*cheA1* and Δ*cheA3* displayed expansion halos indistinguishable from wild-type halos, suggesting that neither CheA1 nor CheA3 is essential for motility under these conditions. In contrast, the Δ*cheA2* mutant exhibited significantly reduced expansion, reaching only 4 cm in diameter over the same period (Fig. 2B, *P* < 0.0001).

To highlight defects in chemotaxis, we examined expansion in a strong chemoattractant gradient. Filter discs saturated with concentrated sSE were placed on one side of the inoculation site, with water-saturated control discs on the opposite side, creating a directional attractant gradient across the plate. Wild-type *B. diazoefficiens* exhibited clear directional bias, with expansion halos extending preferentially toward the sSE source. By day 7, the halo of visible wild-type cells reached the sSE disc but not the control disc, with approximately 60% of their total expansion directed toward sSE (Fig. 2C). Both the Δ*cheA1* and Δ*cheA3* mutant strains displayed similar chemotactic bias to wild type (Fig. 2C, D and F), whereas Δ*cheA2* mutants showed a significant reduction in the directional bias of their expansion halos (Fig. 2C and E, *P* < 0.0001).

To assess whether these results could be explained by differential metabolism of sSE across strains, we performed growth curves comparing wild-type *B. diazoefficiens* with our single *cheA* mutants. We observed nearly identical growth kinetics in minimal AG medium, with all strains exhibiting similar doubling times and reaching maximum optical densities after approximately 40 h. Supplementation of cultures with sSE increased the maximum population density for all strains equally, with no differential effects observed among mutants (Fig. S1A). These results demonstrate that differences in expansion biases toward sSE across genotypes cannot be explained by differences in their growth rates in the presence of sSE.

### The *cheA1* and *cheA2* genes exhibit asymmetric redundancy

Because the Δ*cheA2* mutant retained some expansion bias toward sSE, we hypothesized that che2 is functionally redundant with at least one other che operon. To investigate this, we generated double and triple *cheA* deletion strains. Just as for the single mutants, we found that in liquid media, the double and triple mutants had identical growth kinetics to wild type regardless of the presence of sSE (Fig. S1B).

**Fig 3:**
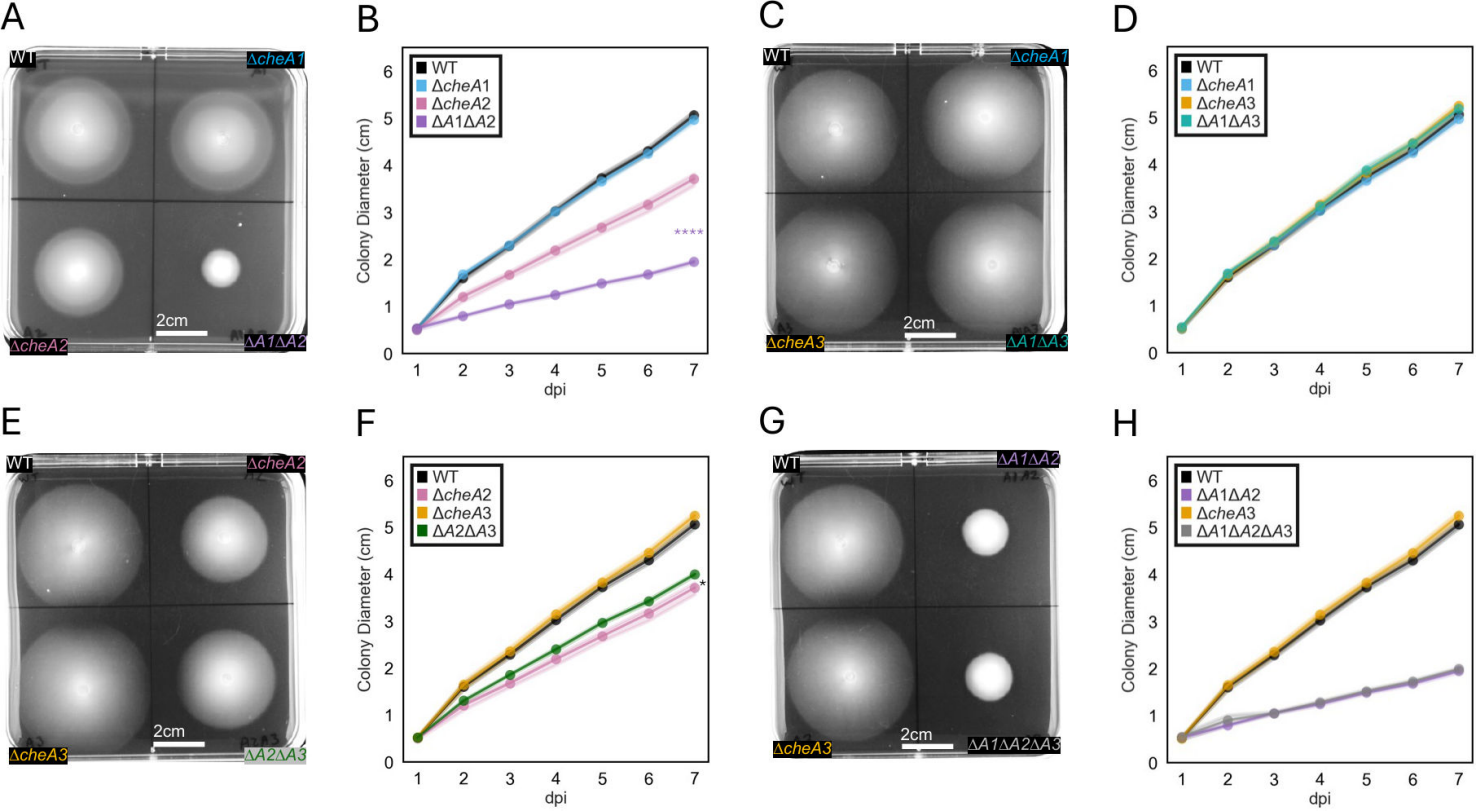
Both *cheA1* and *cheA2*, but not *cheA3*, contribute to colony expansion. Expansion of cells inoculated in semisolid (0.3% agar) minimal AG medium for (**A and B**) Δ*cheA1*Δ*cheA2* compared to Δ*cheA1*, Δ*cheA2*, and WT; (**C and D**) Δ*cheA1*Δ*cheA3* compared to Δ*cheA1*, Δ*cheA3*, and WT; (**E and F**) Δ*cheA2*Δ*cheA3* compared to Δ*cheA2*, Δ*cheA3*, and WT; and (**G and H**) triple knockout strain Δ*cheA1*Δ*cheA2*Δ*cheA3* compared to Δ*cheA1*Δ*cheA,* Δ*cheA3*, and WT. Plate images were collected at 6 dpi. In all line plots, points are means across *N* = 3 plates within a single experiment; shaded bands indicate the range of observed values. Statistical significance was assessed at 7 dpi by one-way ANOVA between WT and each indicated strain: * = P < 0.05, ** = P < 0.01, *** = P < 0.001, **** = P < 0.0001, no asterisk *= P* > 0.05.

We then examined the inoculum expansion rates of the double and triple mutant strains in semisolid agar plates. The double mutant Δ*cheA1*Δ*cheA2* exhibited a significantly more severe defect than the Δ*cheA2* single deletion (*P* ≈ 7.2 × 10⁻⁶), with halos reaching only ~2 cm in diameter after 7 days, representing a 60% reduction compared to wild type (Fig. 3A and B). Loss of *cheA3* yielded strain-dependent effects. The Δ*cheA1*Δ*cheA3* double mutant resembled the wild-type strain (Fig. 3C and D), and the triple mutant Δ*cheA1*Δ*cheA2*Δ*cheA3* was likewise indistinguishable from Δ*cheA1*Δ*cheA2* (Fig. 3G and H; *P* ≈ 0.14 based on one-way ANOVA of 7 dpi values). However, the Δ*cheA2*Δ*cheA3* double mutant expanded slightly more than the Δ*cheA2* single mutant (Fig. 3E and F, *P* = 0.0107), hinting at a potential role of *cheA3* as a negative regulator of chemotaxis when *cheA1* is present.

**Fig 4:**
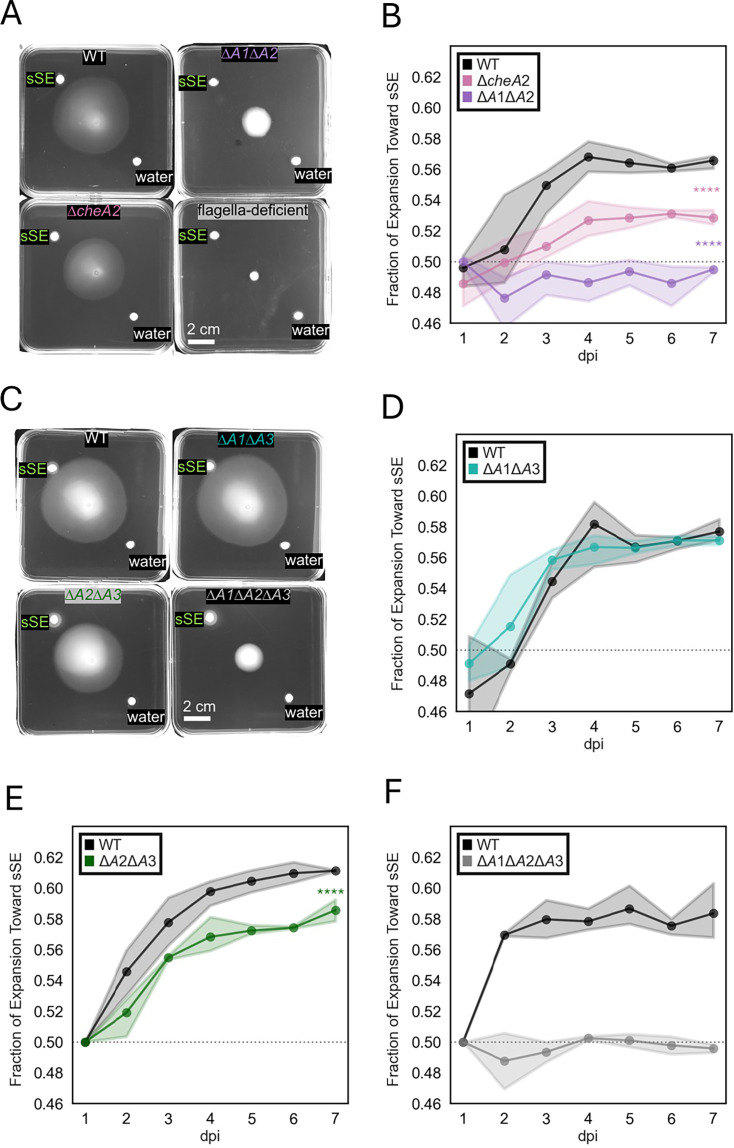
Δ*cheA1*Δ*cheA2* shows no chemotaxis in semisolid media. (**A**) Representative images of expansion halos of WT, Δ*cheA2*, Δ*cheA1*Δ*cheA2*, and the non-motile flagella-deficient strain Δ*fla* (LP 6543 [30]) on semisolid (0.3% agar) minimal AG medium with sSE-saturated and water-saturated discs placed 4 cm from the inoculation point at 7 dpi. (**B**) Fraction of colony expansion toward sSE for WT, Δ*cheA2*, and Δ*cheA1*Δ*cheA2* over 7 days. Data points are means across *N* = 3 plates per strain. Shaded bands indicate the range of observed values, and the dashed horizontal line at 0.5 represents no directional preference. (**C**) Representative plate images at 7 dpi for WT, Δ*cheA1*Δ*cheA3*, Δ*cheA2*Δ*cheA3*, and Δ*cheA1*Δ*cheA2*Δ*cheA3* on plates with sSE-vs-water disc gradients. (**D–F**) Fraction of expansion toward sSE over 7 days for (**D**) Δ*cheA1*Δ*cheA3*, (**E**) Δ*cheA2*Δ*cheA3*, and (**F**) Δ*cheA1*Δ*cheA2*Δ*cheA3*, each compared to WT. Statistical significance in panel **B** and panels **D–F** was determined by one-way ANOVA at 7 dpi for each strain against WT: * = P < 0.05, ** = P < 0.01, *** = P < 0.001, **** = P < 0.0001, no asterisk *= P* > 0*.*05.

To investigate whether the double and triple mutants are defective in chemotaxis, we examined their expansion in the presence of a sSE gradient, as we did for the single deletion strains. The Δ*cheA1*Δ*cheA2* double mutant exhibited no directional preference in its expansion throughout the 7-day observation period (Fig. 4A and B), indicating that this strain is effectively “chemotaxis blind.” The loss of chemotaxis Δ*cheA1*Δ*cheA2* does not appear to result from a total loss of motility in this strain. A flagella-deficient strain (Δ*fla*, from (30); see Table S1) of *B. diazoefficiens* lacking both the subpolar and lateral flagellar gene clusters showed no expansion from the initial inoculation point after 7 days in the presence of sSE gradients (Fig. 4A). This Δ*fla* strain exhibits no growth defect in sSE-supplemented shaking cultures (Fig. S1C), indicating that Δ*fla* can properly metabolize sSE and confirming that the expansion of Δ*cheA1*Δ*cheA2* is flagella-dependent. The loss of chemotaxis in Δ*cheA1*Δ*cheA2* indicates that the che1 and che2 operons exhibit asymmetric redundancy: disrupting che1 (via Δ*cheA1*) has no measurable effect on chemotaxis; disrupting che2 (via Δ*cheA2*) significantly impairs it; and disrupting both abolishes directed movement entirely.

Our finding that Δ*cheA1*Δ*cheA2* cannot chemotax also suggests that che3 does not promote chemotaxis. Plate chemotaxis assays on strains carrying *cheA3* deletions in the presence of sSE gradients support this interpretation, as deletion of *cheA3* did not affect expansional bias towards sSE, regardless of genetic background (Fig. 4C through F). The Δ*cheA1*Δ*cheA3* double mutant displayed a directional bias toward sSE that was indistinguishable from both wild type and the Δ*cheA1* single mutant (Fig. 4D), whereas the triple mutant Δ*cheA1*Δ*cheA2*Δ*cheA3* remained completely chemotaxis-blind (Fig. 4F). The Δ*cheA2*Δ*cheA3* strain showed a reduced chemotactic response (Fig. 4E), similar to that of the Δ*cheA2* single mutant (Fig. 2E), although additional experiments that directly pair these two strains would be necessary to determine whether Δ*cheA2*Δ*cheA3* is a perfect phenocopy of Δ*cheA2*.

### *CheA* mutants retain WT-like motility in aqueous environments

To assess the motility of our *cheA* mutants in aqueous environments, we performed single-cell tracking using fluorescence microscopy (Videos S1 to S5). All single mutants and the Δ*cheA1*Δ*cheA2* double mutant displayed swimming behavior (Fig. S2) and could reach swim speeds comparable to wild-type cells, although all strains also contained a majority of non-motile cells (Table S3). To ensure the high proportion of non-motile cells did not result from a loss of viability, we performed flow cytometry on cell populations prepared identically to those used for microscopy and stained using a LIVE/DEAD kit. Across all genotypes tested, ≥99% cells were viable, and the proportion of propidium iodide-positive cells did not differ significantly between WT and any mutant (Fig. S2A).

**Fig 5:**
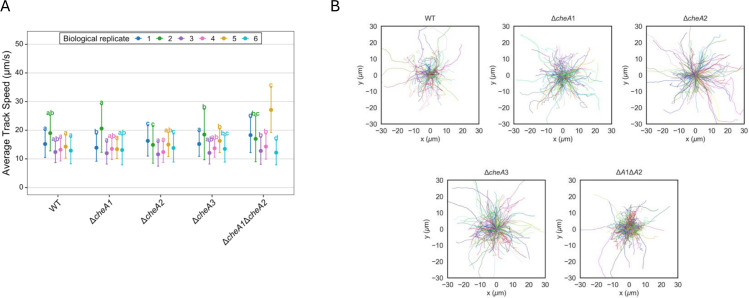
Loss of *cheA* genes does not significantly impact cell swimming behavior in homogeneous aqueous medium. (**A**) Average per-track swimming speeds (μm/s) of single motile cells of WT, Δ*cheA1*, Δ*cheA2*, Δ*cheA3*, and Δ*cheA1*Δ*cheA2* tracked by fluorescence microscopy in homogeneous rich AG medium. Data points represent means ± 1 SD across all motile-cell tracks (after non-motile-cell filtering; see Fig. S3 and Materials and Methods) for six independent biological replicates. Compact-letter annotations denote genotypes with statistically indistinguishable track speeds within each replicate (one-way ANOVA + Tukey’s HSD on per-track values, α = 0.05). (**B**) Representative rose plots showing 200 randomly sampled single-cell trajectories per strain from a single representative biological replicate. Each track was translated so that its origin lies at (0,0); axes are in μm with a fixed range of ± 30 µm. Colors are randomized to distinguish individual tracks within a panel.

Quantitative analysis of swimming speeds from multiple experiments revealed no consistent, statistically significant (*P* < 0.05) differences between strains across biological replicates (Fig. 5; Table 1). We also did not observe consistent, statistically significant differences among strains in the frequencies of run versus tumble events or in the average swimming speeds during run events (Fig. S3; Table 2; Table S3). These single-cell observations confirm that the Δ*cheA1*Δ*cheA2* strain retains fully functional flagella and normal swimming mechanics. Across genotypes, per-replicate average track speeds were compared using one-way ANOVA followed by Tukey’s HSD on the six independent biological replicates. Although occasional pairwise differences emerged within individual replicates (Table 1), no mutant strain differed from WT consistently across the six replicates, and the across-replicate ANOVA on per-replicate means yielded no statistically significant differences (*P* > 0.05).

**TABLE 1:** Loss of *cheA* genes does not reduce cell swimming speeds^*a*^

Expt no.	WT	Δ*cheA1*	Δ*cheA2*	Δ*cheA3*	Δ*cheA1*Δ*cheA2*
1	16.5 ± 4.9^a^	17.1 ± 6.2^a^	17.3 ± 5.1^b^	16.0 ± 4.3^a^	23.0 ± 8.0^c^
2	19.8 ± 5.9^a^	23.4 ± 8.7^b^	17.1 ± 7.0^c^	24.0 ± 9.0^b^	20.5 ± 8.8^a^
3	13.7 ± 4.0^a^	13.7 ± 4.6^a^	13.2 ± 4.6^a^	13.7 ± 4.4^a^	15.1 ± 5.3^b^
4	14.1 ± 4.1^a^	14.7 ± 4.2^a^	13.6 ± 3.8^a^	14.5 ± 3.5^a^	15.8 ± 4.7^b^
5	15.4 ± 4.2^ab^	14.9 ± 3.9^a^	15.9 ± 4.2^b^	16.8 ± 4.1^c^	29.6 ± 6.6^d^
6	15.3 ± 5.5^a^	16.0 ± 6.0^b^	16.0 ± 5.5^b^	15.9 ± 5.5^b^	13.3 ± 4.5^c^
**Avg**	**15.8 ± 4.9**	**16.6 ± 6.0**	**15.5 ± 5.2**	**16.8 ± 5.7**	**19.6 ± 7.0**

^*a*^ Average speeds of single cells of different *cheA* genotypes measured from six independent experiments (*n *> 100 motile cell tracks per experiment). Superscript letters represent statistically indistinguishable groups (*P* > 0.05) as determined by ANOVA. Bold numbers show average values and standard deviation across all experiments.

**TABLE 2:** Run characteristics of WT and *cheA* mutant strains^*a*^

Expt no.	WT	Δ*cheA1*	Δ*cheA2*	Δ*cheA3*	Δ*cheA1*Δ*cheA2*
Mean run-only speed (μm/s)					
1	15.1 ± 5.6^a^	13.7 ± 6.7^b^	16.2 ± 5.7^c^	15.0 ± 5.0^ac^	18.0 ± 6.3^d^
2	19.3 ± 6.7^ab^	20.6 ± 8.8^a^	13.7 ± 7.5^c^	17.9 ± 9.8^bd^	16.3 ± 8.7^cd^
3	12.1 ± 4.9^ab^	10.9 ± 5.7^ac^	10.7 ± 5.9^c^	11.3 ± 5.9^abc^	11.9 ± 7.0^b^
4	13.0 ± 5.8^a^	13.1 ± 5.3^a^	11.6 ± 6.9^a^	13.3 ± 4.4^a^	13.2 ± 6.2^a^
5	13.2 ± 5.6^ab^	12.6 ± 4.7^a^	14.9 ± 5.3^bc^	16.2 ± 4.9^c^	26.9 ± 10.0^d^
6	12.5 ± 7.6^abc^	12.4 ± 7.8^ab^	12.9 ± 7.6^ac^	13.4 ± 7.7^c^	11.7 ± 5.9^b^
**Avg**	**14.9 ± 6.8**	**12.8 ± 7.0**	**13.4 ± 6.9**	**14.4 ± 7.0**	**21.1 ± 11.0**
Mean run length (μm)					
1	15.06 ± 5.60^a^	13.66 ± 6.71^b^	16.16 ± 5.70^c^	14.98 ± 4.97^ac^	18.00 ± 6.25^d^
2	19.32 ± 6.66^ab^	20.56 ± 8.77^a^	13.66 ± 7.48^c^	17.91 ± 9.77^bd^	16.29 ± 8.67^cd^
3	12.07 ± 4.87^ab^	10.90 ± 5.73^ac^	10.69 ± 5.86^c^	11.29 ± 5.87^abc^	11.86 ± 6.99^b^
4	12.99 ± 5.84^a^	13.07 ± 5.33^a^	11.58 ± 6.92^a^	13.30 ± 4.43^a^	13.21 ± 6.24^a^
5	13.25 ± 5.64^ab^	12.58 ± 4.74^a^	14.90 ± 5.29^bc^	16.16 ± 4.89^c^	26.90 ± 10.05^d^
6	12.55 ± 7.62^abc^	12.39 ± 7.75^ab^	12.90 ± 7.58^ac^	13.38 ± 7.75^c^	11.67 ± 5.92^b^
**Avg**	**14.90 ± 6.83**	**12.82 ± 7.05**	**13.40 ± 6.86**	**14.37 ± 6.97**	**21.15 ± 10.97**
Mean run duration (s)					
1	0.44 ± 0.42^a^	0.25 ± 0.27^b^	0.54 ± 0.43^c^	0.59 ± 0.46^c^	0.70 ± 0.56^d^
2	0.31 ± 0.28^ab^	0.27 ± 0.23^a^	0.16 ± 0.21^c^	0.12 ± 0.12^c^	0.36 ± 0.35^b^
3	0.35 ± 0.36^a^	0.25 ± 0.32^b^	0.30 ± 0.34^ab^	0.22 ± 0.26^b^	0.29 ± 0.40^ab^
4	0.30 ± 0.29^ab^	0.29 ± 0.30^a^	0.20 ± 0.23^a^	0.24 ± 0.22^a^	0.38 ± 0.50^b^
5	0.30 ± 0.34^a^	0.42 ± 0.46^b^	0.61 ± 0.48^c^	0.54 ± 0.41^c^	0.23 ± 0.39^d^
6	0.18 ± 0.26^a^	0.17 ± 0.22^a^	0.22 ± 0.29^a^	0.20 ± 0.25^a^	0.58 ± 0.69^b^
**Avg**	**0.33 ± 0.35**	**0.24 ± 0.29**	**0.35 ± 0.39**	**0.30 ± 0.34**	**0.36 ± 0.50**
Run-to-non-run time ratio					
1	3.50 ± 3.51^a^	1.83 ± 2.33^b^	4.50 ± 3.83^c^	4.81 ± 3.97^c^	5.80 ± 4.79^d^
2	2.55 ± 2.41^a^	1.97 ± 1.94^b^	0.96 ± 1.67^c^	0.70 ± 0.96^c^	2.84 ± 3.04^a^
3	2.73 ± 3.13^a^	1.89 ± 2.88^b^	2.22 ± 2.98^ab^	1.63 ± 2.20^b^	2.14 ± 3.48^ab^
4	2.27 ± 2.42^abc^	2.27 ± 2.61^ab^	1.45 ± 1.95^ac^	1.75 ± 1.90^c^	2.74 ± 4.00^b^
5	2.34 ± 2.94^a^	3.41 ± 3.96^b^	5.06 ± 4.11^c^	4.45 ± 3.39^c^	1.65 ± 3.24^d^
6	1.29 ± 2.25^ab^	1.17 ± 1.86^a^	1.59 ± 2.45^b^	1.35 ± 2.09^ab^	4.69 ± 5.88^c^
**Avg**	**2.54 ± 3.00**	**1.80 ± 2.56**	**2.70 ± 3.39**	**2.31 ± 2.95**	**2.72 ± 4.28**

^*a*^ Mean run speed (μm/s), mean run length (μm), mean run duration (s), and run-to-non-run time ratio for single motile cells of each genotype, measured from six independent experiments (*n* > 100 identified run events per experiment). A run was defined as a sequence of consecutive frames in which the cell moved in a persistent direction (see Materials and Methods). Values are mean ± SD.

We also sought to determine the chemotaxis behavior of our strains in aqueous media. However, despite the robust chemotaxis we observed in soft agar assays, analysis of individual *B. diazoefficiens* cells in standard microscopy-based chemotaxis assays (46, 48) revealed no detectable chemotactic behavior in liquid culture. When exposed to gradients of sSE, rich AG medium, or 10 mM glutamate, wild-type cells showed no directional bias compared to control conditions (Fig. S4).

### Coding sequences do not determine *cheA* functional hierarchy

**Fig 6:**
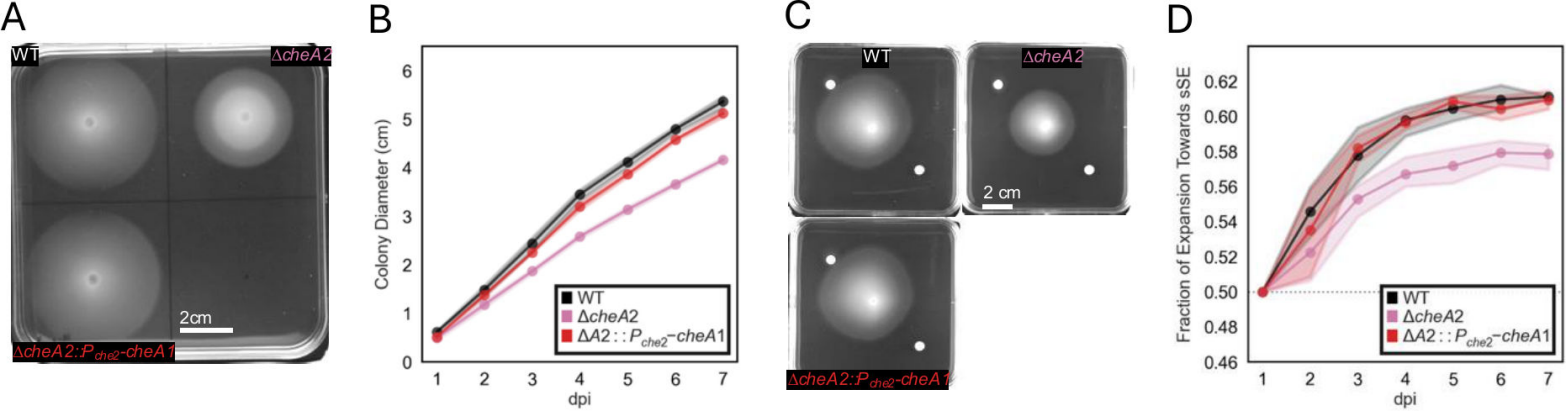
Expression of *cheA1* from the che2 operon restores the Δ*cheA2* phenotype to wild type. (**A**) Representative images at 7 dpi of expansion halos for WT, Δ*cheA2*, and Δ*cheA2*::P*che2-cheA1* on semisolid minimal AG medium. (**B**) Expansion-halo diameter over 7 days for the strains in panel A. (**C**) Representative plate images at 7 dpi of the same three strains on semisolid plates containing sSE-saturated and water-saturated filter discs placed 4 cm from the inoculation point. (**D**) Fraction of expansion toward sSE over 7 days for the strains in panel C; means across *N* = 3 plates per strain, shaded bands indicate the range of observed values; dashed line at 0.5 = no directional preference. A one-way ANOVA at 7 dpi showed that Δ*cheA2*::P*che2-cheA1* is not significantly different from WT in expansion diameter (one-way ANOVA at 7 dpi: WT vs Δ*cheA2*::P*_che2_-cheA1 P* ≈ 0.094) or in directional bias (WT vs Δ*cheA2::P_che2_-cheA1 P* ≈ 0.71). In contrast, WT vs Δ*cheA2* is significantly different in diameter at 7 dpi (*P* ≈ 2.6 × 10⁻⁶).

Given that CheA1 and CheA2 share >99% amino acid sequence identity (Fig. 1B) yet display asymmetric functional importance, we hypothesized that their differential contributions to chemotaxis arise from differences in gene regulation or operon composition. This hypothesis is consistent with our observation that *cheA2* is expressed at ~30% higher levels than *cheA1* in minimal medium cultures and ~10% higher than *cheA1* after sSE induction (Fig. 1C). To test this hypothesis, we generated a complementation strain in which the native *cheA2* gene was replaced with *cheA1*, placing *cheA1* under control of the che2 promoter (Δ*cheA2*::P_che2_-*cheA1*). Remarkably, this strain fully restored wild-type chemotactic behavior in semisolid sSE gradient assays, with expansion patterns and directional bias indistinguishable from the parental wild-type strain (Fig. 6). Conversely, placing the *cheA2* coding sequence under control of the che1 promoter in a Δ*cheA1* background (Δ*cheA1*::P_che1_-*cheA2*) did not increase expansion bias beyond that of the WT (Fig. S5A and B). These patterns were recapitulated in the double deletion background: insertion of cheA1 coding sequence under control of the che2 promoter (Δ*cheA1*Δ*cheA2*::P_che2_-*cheA1*) fully restored wild-type expansion, whereas insertion of cheA2 under control of the che1 promoter (Δ*cheA1*Δ*cheA2*::P_che1_-*cheA2*) resulted in a Δ*cheA2* phenocopy (Fig. S5C and D). Together, these reciprocal swap experiments demonstrate that the functional hierarchy between che1 and che2 is not determined by the coding sequence of their respective *cheA* genes.

## DISCUSSION

Robust chemotaxis is correlated with higher fitness in plant-beneficial soil bacteria and is an important consideration in the engineering of agricultural inoculants. In this study, we provide the first genetic analysis of chemotaxis in the soybean inoculant *B. diazoefficiens* USDA110. We found that, of the three copies of *cheA* in this organism, only *cheA1* and *cheA2* contribute positively to chemotaxis toward soybean seed exudates in soft agar. Because deleting a *cheA* gene typically disrupts downstream *che* genes within the same operon, our results reflect the relative contributions of the operons as a whole rather than the specific roles of each *cheA*.

Although strains with disruptions in either the che1 or che2 operon alone maintain some chemotactic ability, the combined disruption of both operons abolishes directed movement toward sSE in soft agar assays. This suggests a functional redundancy between che1 and che2 that may enable more robust chemotaxis in the native environment, ensuring that bacteria can respond to host signals even if one pathway is compromised. Our observations that *cheA1* expressed at *the cheA2* locus (Δ*cheA2*::P_che2_-*cheA1*) can restore wild-type chemotaxis, and that *cheA2* is expressed at a higher level than *cheA1*, suggest that their relative contributions result from differences in regulation or downstream signaling partners rather than their protein sequences. We do not yet know what factors drive this differential regulation, nor the extent to which the relative induction of each operon is related to the available nutrient sources. Future studies will be needed to determine whether differential che operon induction is tuned by their responsiveness to different attractant compounds or by their relative affinities for shared transcriptional regulators.

We did not observe a chemotaxis defect for *cheA3* deletion mutants. Although this could stem from a mismatch between the chemoattractants in AG medium and sSE and those in the native environment, we think this is unlikely. Unlike CheA1 and CheA2, CheA3 lacks the CheY-binding P2 domain essential for phosphotransfer to the response regulator. This structural difference should preclude CheA3 from directly controlling flagellar rotation through the canonical phosphorelay pathway (49). It is possible that this truncated CheA3 protein can attenuate signals from CheA1 and CheA2 by competing for binding to methyl-accepting chemotaxis proteins (MCPs). The slight but statistically significant (*P* = 0.0107) enhancement of motility upon *cheA3* deletion in the Δ*cheA2* background provides some support for this model, though further biochemical studies are needed to test this hypothesis.

Our observation that *cheA* deletion mutants retain WT-like swimming speeds in aqueous medium is notable given findings in other bacteria with dual-flagellar systems. In *Rhizobium leguminosarum*, loss of the organism’s *cheA* genes increases swimming speeds due to reduced tumble frequency (21). In contrast, in *A. brasilense*, loss of some *cheA* genes moderately affects swimming speeds, whereas loss of other *cheA* loci has no effect (50, 51). In more distantly related dual-flagella organisms, swimming speeds have sometimes been reported as being affected by loss of chemotaxis machinery (e.g., *Vibrio harveyi* [52]) and sometimes not (e.g., *Vibrio parahaemolyticus* (53, 54]). Future experiments will be needed to understand why the degree of coupling between flagellar rotation speeds, activation of *che* gene signaling, and the number of types of flagella is so variable across species.

We were surprised to observe that single *B. diazoefficiens* USDA110 cells do not chemotax effectively in liquid environments under conditions used to induce aqueous chemotaxis in *Vibrio* spp. and other rhizobia. We propose two potential explanations for the discrepancy between our population-level soft agar chemotaxis assays and single-cell aqueous chemotaxis experiments. One possibility is that aqueous chemotaxis only occurs in response to chemoattractants that are not present in sSE or AG medium, or that swimming bias toward these compounds is difficult to capture in the lab. For example, soil bacteria are known to require more extensive starvation and a much narrower, lower concentration range to induce chemotaxis than biomedical model species. *B. diazoefficiens* may be at an extreme end of this continuum or may only exhibit detectable single-cell chemotaxis on very long timescales. Another possible explanation is that in our strain of *B. diazoefficiens* USDA110, chemotaxis simply does not occur in the liquid conditions used in this study. The physical constraints of moving through a semisolid matrix might enable detection of chemical gradients through mechanisms that are inactive in purely aqueous conditions. There is already evidence that the *B. diazoefficiens* USDA110 flagellar motility systems are engaged in different environments, with the lateral flagella dominating in movement on or near surfaces (55). If the *che* pathways of *B. diazoefficiens* USDA110 primarily regulate lateral flagella-driven surface motility, they may be less active in an open aqueous environment. Given that *B. diazoefficiens* USDA110 naturally resides near soil particles and plant tissues, it may be that robust chemotaxis through open aqueous environments makes an insignificant contribution to fitness in its native ecological context.

Regardless of the underlying mechanism, our data indicate that chemoattractant responses of *B. diazoefficiens* USDA110 in liquid are distinct from previously studied soil bacterial models. Future work examining the relative importance of these systems in more complex soil-like environments will be important to understand how *B. diazoefficiens* chemotaxis systems are integrated in real-world agricultural settings. Understanding how *B. diazoefficiens* USDA110 responds to soils could benefit agriculture, including by optimizing the timing of inoculation relative to irrigation or by improving carrier materials for bacterial inoculant delivery. Such improvements could enhance the robustness of plant-microbe interactions across varying soil conditions, an important consideration as climate change leads to more variable precipitation patterns in agricultural regions.

## SUPPLEMENTAL MATERIAL

Supplemental figures aem.00928-26-s0001.pdf Fig. S1 to S5.

Supplemental legends aem.00928-26-s0002.docx Descriptive legends for Videos S1 to S5.

Supplemental tables aem.00928-26-s0003.docx Tables S1 to S4.

Video S1 aem.00928-26-s0004.mov Representative swimming wild-type *B. diazoefficiens* cells.

Video S2 aem.00928-26-s0005.mov Representative swimming Δ*cheA1* cells.

Video S3 aem.00928-26-s0006.mov Representative swimming Δ*cheA2* cells.

Video S4 aem.00928-26-s0007.mov Representative swimming Δ*cheA3* cells.

Video S5 aem.00928-26-s0008.mov Representative swimming Δ*cheA1*Δ*cheA2* cells.

ASM does not own the copyrights to Supplemental Material that may be linked to, or accessed through, an article. The authors have granted ASM a non-exclusive, world-wide license to publish the Supplemental Material files. Please contact the corresponding author directly for reuse.

## References

[1] Iqbal S, Begum F, Nguchu BA, Claver UP, Shaw P. 2025. The invisible architects: microbial communities and their transformative role in soil health and global climate changes. Environ Microbiome 20:36. doi:10.1186/s40793-025-00694-6 40133952 PMC11938724

[2] Mendoza-Suárez M, Andersen SU, Poole PS, Sánchez-Cañizares C. 2021. Competition, nodule occupancy, and persistence of inoculant strains: key factors in the Rhizobium-legume symbioses. Front Plant Sci 12:690567. doi:10.3389/fpls.2021.690567 34489993 PMC8416774

[3] Yang P, van Elsas JD. 2018. Mechanisms and ecological implications of the movement of bacteria in soil. Appl Soil Ecol 129:112–120. doi:10.1016/j.apsoil.2018.04.014

[4] Raina J-B, Fernandez V, Lambert B, Stocker R, Seymour JR. 2019. The role of microbial motility and chemotaxis in symbiosis. Nat Rev Microbiol 17:284–294. doi:10.1038/s41579-019-0182-9 30923350

[5] Buchan A, Crombie B, Alexandre GM. 2010. Temporal dynamics and genetic diversity of chemotactic-competent microbial populations in the rhizosphere. Environ Microbiol 12:3171–3184. doi:10.1111/j.1462-2920.2010.02290.x 20629701

[6] Poole P, Ramachandran V, Terpolilli J. 2018. Rhizobia: from saprophytes to endosymbionts. Nat Rev Microbiol 16:291–303. doi:10.1038/nrmicro.2017.171 29379215

[7] Catlow HY, Glenn AR, Dilworth MJ. 1990. Does rhizobial motility affect its ability to colonize along the legume root? Soil Biol Biochem 22:573–575. doi:10.1016/0038-0717(90)90196-7

[8] Aroney STN, Poole PS, Sánchez-Cañizares C. 2021. Rhizobial chemotaxis and motility systems at work in the soil. Front Plant Sci 12:725338. doi:10.3389/fpls.2021.725338 34512702 PMC8429497

[9] Ramoneda J, Fan K, Lucas JM, Chu H, Bissett A, Strickland MS, Fierer N. 2024. Ecological relevance of flagellar motility in soil bacterial communities. ISME J 18:wrae067. doi:10.1093/ismejo/wrae067 38648266 PMC11095265

[10] Alexandre G. 2025. Movement of bacteria in the soil and the rhizosphere. Appl Environ Microbiol 91:e0024625. doi:10.1128/aem.00246-25 40938096 PMC12542687

[11] Scharf BE, Hynes MF, Alexandre GM. 2016. Chemotaxis signaling systems in model beneficial plant-bacteria associations. Plant Mol Biol 90:549–559. doi:10.1007/s11103-016-0432-4 26797793

[12] He K, Marden JN, Quardokus EM, Bauer CE, Viollier PH. 2013. Phosphate flow between hybrid histidine kinases CheA₃ and CheS₃ controls Rhodospirillum centenum cyst formation. PLoS Genet 9:e1004002. doi:10.1371/journal.pgen.1004002 24367276 PMC3868531

[13] Liu W, Sun Y, Shen R, Dang X, Liu X, Sui F, Li Y, Zhang Z, Alexandre G, Elmerich C, Xie Z. 2018. A chemotaxis-like pathway of Azorhizobium caulinodans controls flagella-driven motility, which regulates biofilm formation, exopolysaccharide biosynthesis, and competitive nodulation. Mol Plant Microbe Interact 31:737–749. doi:10.1094/MPMI-12-17-0290-R 29424664

[14] Ganusova EE, Vo LT, Mukherjee T, Alexandre G. 2021. Multiple CheY proteins control surface-associated lifestyles of Azospirillum brasilense. Front Microbiol 12:664826. doi:10.3389/fmicb.2021.664826 33968002 PMC8100600

[15] Ganusova EE, Russell MH, Patel S, Seats T, Alexandre G. 2024. An Azospirillum brasilense chemoreceptor that mediates nitrate chemotaxis has conditional roles in the colonization of plant roots. Appl Environ Microbiol 90:e0076024. doi:10.1128/aem.00760-24 38775579 PMC11218637

[16] O’Neal L, Akhter S, Alexandre G. 2019. A PilZ-containing chemotaxis receptor mediates oxygen and wheat root sensing in Azospirillum brasilense. Front Microbiol 10:312. doi:10.3389/fmicb.2019.00312 30881352 PMC6406031

[17] Liu X, Liu W, Sun Y, Xia C, Elmerich C, Xie Z. 2018. A cheZ-like gene in Azorhizobium caulinodans is a key gene in the control of chemotaxis and colonization of the host plant. Appl Environ Microbiol 84:e01827-17. doi:10.1128/AEM.01827-17 29150498 PMC5772239

[18] Liu W, Bai X, Li Y, Min J, Kong Y, Hu X. 2020. CheY1 and CheY2 of Azorhizobium caulinodans ORS571 regulate chemotaxis and competitive colonization with the host plant. Appl Environ Microbiol 86:e00599-20. doi:10.1128/AEM.00599-20 32471918 PMC7376556

[19] Matilla MA, Krell T. 2018. The effect of bacterial chemotaxis on host infection and pathogenicity. FEMS Microbiol Rev 42. doi:10.1093/femsre/fux052 29069367

[20] Miller LD, Yost CK, Hynes MF, Alexandre G. 2007. The major chemotaxis gene cluster of Rhizobium leguminosarum bv. viciae is essential for competitive nodulation. Mol Microbiol 63:348–362. doi:10.1111/j.1365-2958.2006.05515.x 17163982

[21] Aroney STN, Pini F, Kessler C, Poole PS, Sánchez-Cañizares C. 2024. The motility and chemosensory systems of Rhizobium leguminosarum, their role in symbiosis, and link to PTS^Ntr^ regulation. Environ Microbiol 26:e16570. doi:10.1111/1462-2920.16570 38216524 PMC7617929

[22] Turnbull GA, Morgan JAW, Whipps JM, Saunders JR. 2001. The role of bacterial motility in the survival and spread of Pseudomonas fluorescens in soil and in the attachment and colonisation of wheat roots. FEMS Microbiol Ecol 36:21–31. doi:10.1111/j.1574-6941.2001.tb00822.x 11377770

[23] Medici IF, Bartrolí L, Guaimas FF, Fulgenzi FR, Molina CL, Sánchez IE, Comerci DJ, Mongiardini E, Soler-Bistué A. 2024. The distinct cell physiology of Bradyrhizobium at the population and cellular level. BMC Microbiol 24:129. doi:10.1186/s12866-024-03272-x 38643099 PMC11031950

[24] Althabegoiti MJ, López-García SL, Piccinetti C, Mongiardini EJ, Pérez-Giménez J, Quelas JI, Perticari A, Lodeiro AR. 2008. Strain selection for improvement of Bradyrhizobium japonicum competitiveness for nodulation of soybean. FEMS Microbiol Lett 282:115–123. doi:10.1111/j.1574-6968.2008.01114.x 18336548

[25] López-García SL, Vázquez TEE, Favelukes G, Lodeiro AR. 2002. Rhizobial position as a main determinant in the problem of competition for nodulation in soybean. Environ Microbiol 4:216–224. doi:10.1046/j.1462-2920.2002.00287.x 12010128

[26] López‐García SL, Perticari A, Piccinetti C, Ventimiglia L, Arias N, De Battista JJ, Althabegoiti MJ, Mongiardini EJ, Pérez‐Giménez J, Quelas JI, Lodeiro AR. 2009. In-furrow inoculation and selection for higher motility enhances the efficacy of Bradyrhizobium japonicum nodulation. Agron J 101:357–363. doi:10.2134/agronj2008.0155x

[27] Althabegoiti MJ, Covelli JM, Pérez-Giménez J, Quelas JI, Mongiardini EJ, López MF, López-García SL, Lodeiro AR. 2011. Analysis of the role of the two flagella of Bradyrhizobium japonicum in competition for nodulation of soybean. FEMS Microbiol Lett 319:133–139. doi:10.1111/j.1574-6968.2011.02280.x 21470300

[28] López-Farfán D, Reyes-Darias JA, Matilla MA, Krell T. 2019. Concentration dependent effect of plant root exudates on the chemosensory systems of Pseudomonas putida KT2440. Front Microbiol 10:78. doi:10.3389/fmicb.2019.00078 30761113 PMC6363813

[29] Wadisirisuk P, Danso SK, Hardarson G, Bowen GD. 1989. Influence of Bradyrhizobium japonicum location and movement on nodulation and nitrogen fixation in soybeans. Appl Environ Microbiol 55:1711–1716. doi:10.1128/aem.55.7.1711-1716.1989 16347964 PMC202939

[30] Quelas JI, Althabegoiti MJ, Jimenez-Sanchez C, Melgarejo AA, Marconi VI, Mongiardini EJ, Trejo SA, Mengucci F, Ortega-Calvo J-J, Lodeiro AR. 2016. Swimming performance of Bradyrhizobium diazoefficiens is an emergent property of its two flagellar systems. Sci Rep 6:23841. doi:10.1038/srep23841 27053439 PMC4823718

[31] Cogo C, Pérez-Giménez J, Rajeswari CB, Luna MF, Lodeiro AR. 2018. Induction by Bradyrhizobium diazoefficiens of different pathways for growth in d-mannitol or l-arabinose leading to pronounced differences in CO_2_ fixation, O_2_ consumption, and lateral-flagellum production. Front Microbiol 9:1189. doi:10.3389/fmicb.2018.01189 29922265 PMC5996035

[32] Mongiardini EJ, Quelas JI, Dardis C, Althabegoiti MJ, Lodeiro AR. 2017. Transcriptional control of the lateral-flagellar genes of Bradyrhizobium diazoefficiens. J Bacteriol 199:e00253-17. doi:10.1128/JB.00253-17 28533217 PMC5512216

[33] Dardis C, Quelas JI, Mengucci F, Althabegoiti MJ, Lodeiro AR, Mongiardini EJ. 2021. Dual control of flagellar synthesis and exopolysaccharide production by FlbD-FliX class II regulatory proteins in Bradyrhizobium diazoefficiens. J Bacteriol 203:e00403-20. doi:10.1128/JB.00403-20 PMC8088514 33468586

[34] Sandhu AK, Brown MR, Subramanian S, Brözel VS. 2023. Bradyrhizobium diazoefficiens USDA 110 displays plasticity in the attachment phenotype when grown in different soybean root exudate compounds. Front Microbiol 14:1190396. doi:10.3389/fmicb.2023.1190396 37275139 PMC10233038

[35] McDermott TR, Graham PH. 1989. Bradyrhizobium japonicum inoculant mobility, nodule occupancy, and acetylene reduction in the soybean root system. Appl Environ Microbiol 55:2493–2498. doi:10.1128/aem.55.10.2493-2498.1989 16348026 PMC203110

[36] Barbour WM, Hattermann DR, Stacey G. 1991. Chemotaxis of Bradyrhizobium japonicum to soybean exudates. Appl Environ Microbiol 57:2635–2639. doi:10.1128/aem.57.9.2635-2639.1991 1768137 PMC183632

[37] Typas A, Sourjik V. 2015. Bacterial protein networks: properties and functions. Nat Rev Microbiol 13:559–572. doi:10.1038/nrmicro3508 26256789

[38] Bi S, Sourjik V. 2018. Stimulus sensing and signal processing in bacterial chemotaxis. Curr Opin Microbiol 45:22–29. doi:10.1016/j.mib.2018.02.002 29459288

[39] Gumerov VM, Andrianova EP, Zhulin IB. 2021. Diversity of bacterial chemosensory systems. Curr Opin Microbiol 61:42–50. doi:10.1016/j.mib.2021.01.016 33684668 PMC8727887

[40] Kaneko T, Nakamura Y, Sato S, Minamisawa K, Uchiumi T, Sasamoto S, Watanabe A, Idesawa K, Iriguchi M, Kawashima K, Kohara M, Matsumoto M, Shimpo S, Tsuruoka H, Wada T, Yamada M, Tabata S. 2002. Complete genomic sequence of nitrogen-fixing symbiotic bacterium Bradyrhizobium japonicum USDA110. DNA Res 9:189–197. doi:10.1093/dnares/9.6.189 12597275

[41] Gumerov VM, Ulrich LE, Zhulin IB. 2024. MiST 4.0: a new release of the microbial signal transduction database, now with a metagenomic component. Nucleic Acids Res 52:D647–D653. doi:10.1093/nar/gkad847 37791884 PMC10767990

[42] King WL, Bell TH. 2022. Can dispersal be leveraged to improve microbial inoculant success? Trends Biotechnol 40:12–21. doi:10.1016/j.tibtech.2021.04.008 33972105

[43] Atieno M, Lesueur D. 2019. Opportunities for improved legume inoculants: enhanced stress tolerance of rhizobia and benefits to agroecosystems. Symbiosis 77:191–205. doi:10.1007/s13199-018-0585-9

[44] Basile LA, Lepek VC. 2021. Legume–rhizobium dance: an agricultural tool that could be improved? Microb Biotechnol 14:1897–1917. doi:10.1111/1751-7915.13906 34318611 PMC8449669

[45] Simon R, O’Connell M, Labes M, Pühler A. 1986. Plasmid vectors for the genetic analysis and manipulation of rhizobia and other gram-negative bacteria. Methods Enzymol 118:640–659. doi:10.1016/0076-6879(86)18106-7 3005803

[46] Grognot M, Nam JW, Elson LE, Taute KM. 2023. Physiological adaptation in flagellar architecture improves Vibrio alginolyticus chemotaxis in complex environments. Proc Natl Acad Sci USA 120:e2301873120. doi:10.1073/pnas.2301873120 37579142 PMC10450658

[47] Pottash AE, McKay R, Virgile CR, Ueda H, Bentley WE. 2017. TumbleScore: run and tumble analysis for low frame-rate motility videos. BioTechniques 62:31–36. doi:10.2144/000114493 28118813

[48] Barbour KM, Barrón-Sandoval A, Walters KE, Martiny JBH. 2023. Towards quantifying microbial dispersal in the environment. Environ Microbiol 25:137–142. doi:10.1111/1462-2920.16270 36308707 PMC10100412

[49] Berry MA, Andrianova EP, Zhulin IB. 2024. Diverse domain architectures of CheA histidine kinase, a central component of bacterial and archaeal chemosensory systems. Microbiol Spectr 12:e0346423. doi:10.1128/spectrum.03464-23 38038435 PMC10782961

[50] Bible A, Russell MH, Alexandre G. 2012. The Azospirillum brasilense Che1 chemotaxis pathway controls swimming velocity, which affects transient cell-to-cell clumping. J Bacteriol 194:3343–3355. doi:10.1128/JB.00310-12 22522896 PMC3434747

[51] Mukherjee T, Kumar D, Burriss N, Xie Z, Alexandre G. 2016. Azospirillum brasilense chemotaxis depends on two signaling pathways regulating distinct motility parameters. J Bacteriol 198:1764–1772. doi:10.1128/JB.00020-16 27068592 PMC4886762

[52] Xu X, Li H, Qi X, Chen Y, Qin Y, Zheng J, Jiang X. 2021. cheA, cheB, cheR, cheV, and cheY are involved in regulating the adhesion of Vibrio harveyi. Front Cell Infect Microbiol 10. doi:10.3389/fcimb.2020.591751 PMC7887938 33614522

[53] Brenzinger S, Pecina A, Mrusek D, Mann P, Völse K, Wimmi S, Ruppert U, Becker A, Ringgaard S, Bange G, Thormann KM. 2018. ZomB is essential for flagellar motor reversals in Shewanella putrefaciens and Vibrio parahaemolyticus. Mol Microbiol 109:694–709. doi:10.1111/mmi.14070 29995998

[54] Sar N, McCarter L, Simon M, Silverman M. 1990. Chemotactic control of the two flagellar systems of Vibrio parahaemolyticus. J Bacteriol 172:334–341. doi:10.1128/jb.172.1.334-341.1990 2294089 PMC208437

[55] Carrillo-Mora JP, Monteiro MP, Lodeiro AR, Marconi VI, Cordero ML. 2025. Damage and recovery of flagella in soil bacteria exposed to shear within long microchannels. arXiv. doi:10.48550/arXiv.2410.10932

