# Supplementary material for: Characterization of chemotaxis in soybean symbiont Bradyrhizobium diazoefficiens
Source: Appl Environ Microbiol. 2026 Jun 24;92(7):e00928-26. doi: 10.1128/aem.00928-26 (PMC13390405; doi:10.1128/aem.00928-26)
Supplement: Supplemental figures — Fig. S1 to S5. [file aem.00928-26-s0001.pdf]

## SUPPLEMENTAL FIGURES

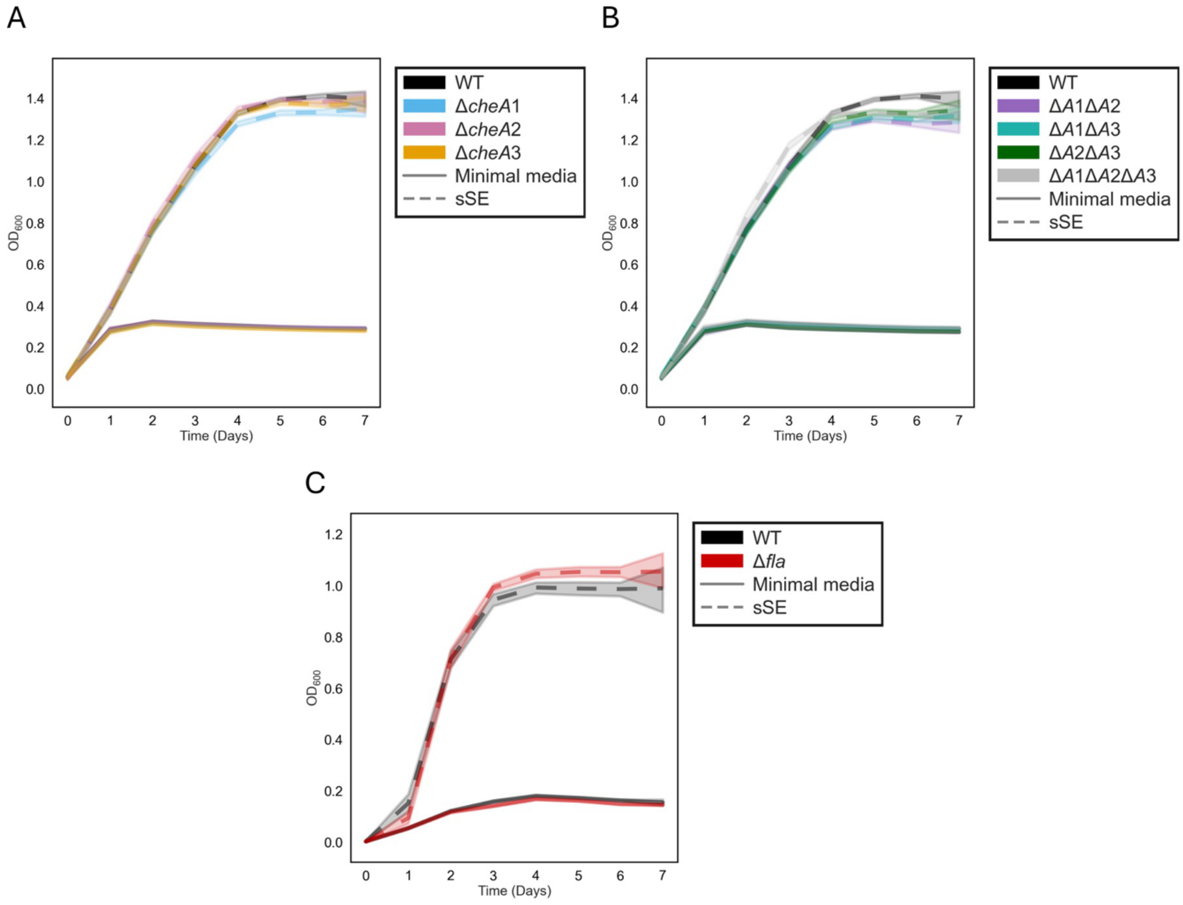


**Figure S1. Loss of *cheA* genes does not impact cell growth in response to soybean seed exudate (sSE) (A)** Liquid growth curves ($OD_{600}$ vs time, in days) of WT and the three single *cheA* deletion mutants (Δ*cheA1*, Δ*cheA2*, Δ*cheA3*) grown in minimal AG medium alone (solid lines) or supplemented with a 1:10 dilution of 10× sSE (dashed lines). **(B)** Growth curves of double and triple deletion mutants Δ*cheA1*Δ*cheA2*, Δ*cheA1*Δ*cheA3*, Δ*cheA2*Δ*cheA3*, and Δ*cheA1*Δ*cheA2*Δ*cheA3* compared with WT. **(C)** Growth curve of the flagella-deficient mutant Δ*fla* compared to WT.

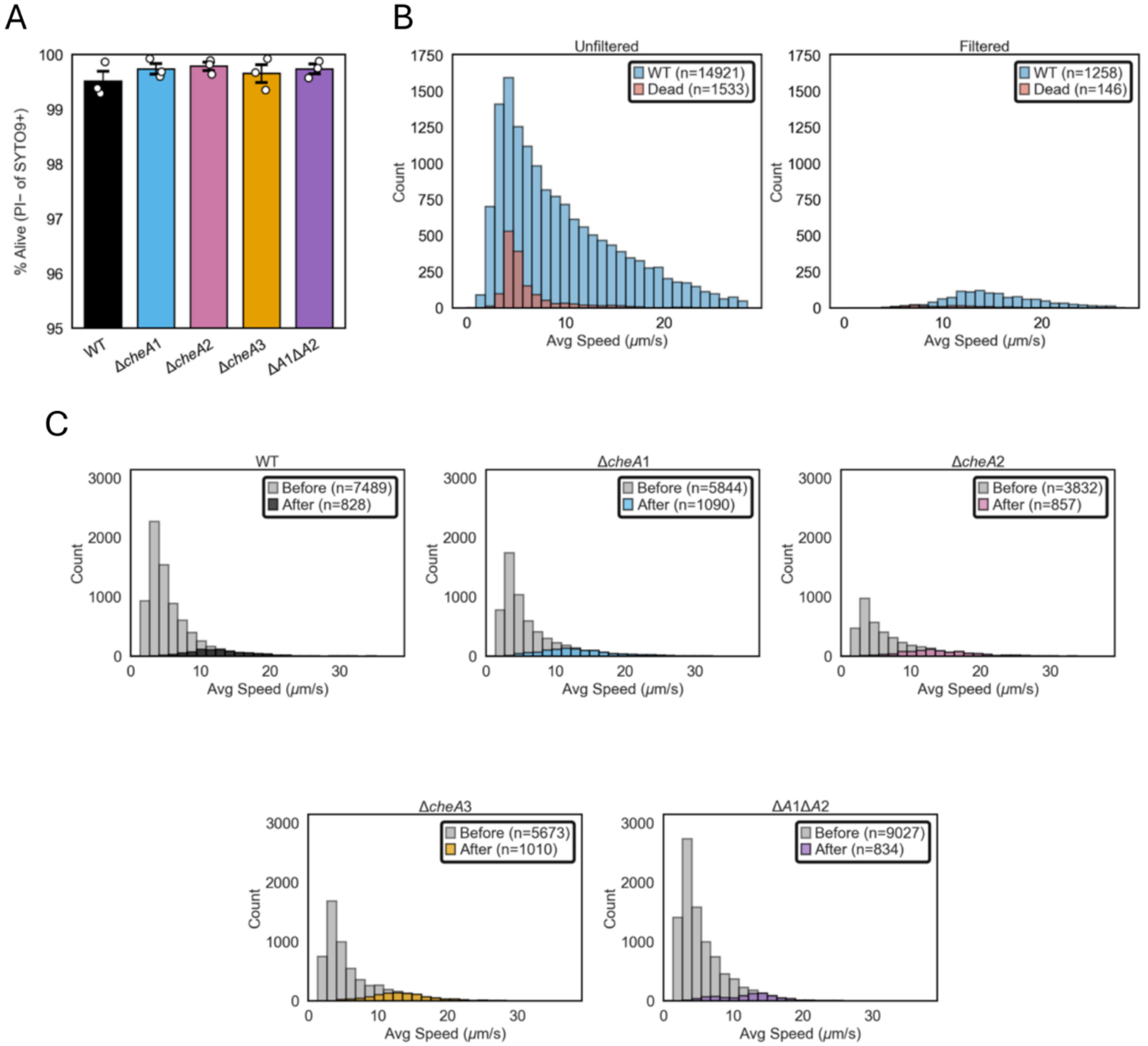


**Figure S2. Filtering out non-motile cells is necessary for accurate representations of cell swimming speeds. (A)** Cell viability by flow cytometry of cells stained with SYTO9/propidium iodide (PI) for live/dead for quantification. Bars show the average percentage of SYTO $9^+$ events that were also $PI^-$. Error bars indicate standard error across 3 technical replicates. **(B)** Histograms of average per-track swimming speeds (μm/s) for a single representative WT replicate compared with formaldehyde-fixed dead-cell controls. Left: unfiltered tracks. Right: tracks remaining after the non-motile-cell filter is applied (a track is removed if no 5-frame sliding window contains ≥ 50 pixels of summed displacement). Bin edges are matched between panels; n values are indicated in the legend. **(C)** Per-strain histograms of average track speeds for cells of WT, Δ*cheA1*, Δ*cheA2*, Δ*cheA3*, and Δ*cheA1*Δ*cheA2* from one representative biological replicate, shown before (grey) and after (colored) the non-motile-cell filter.

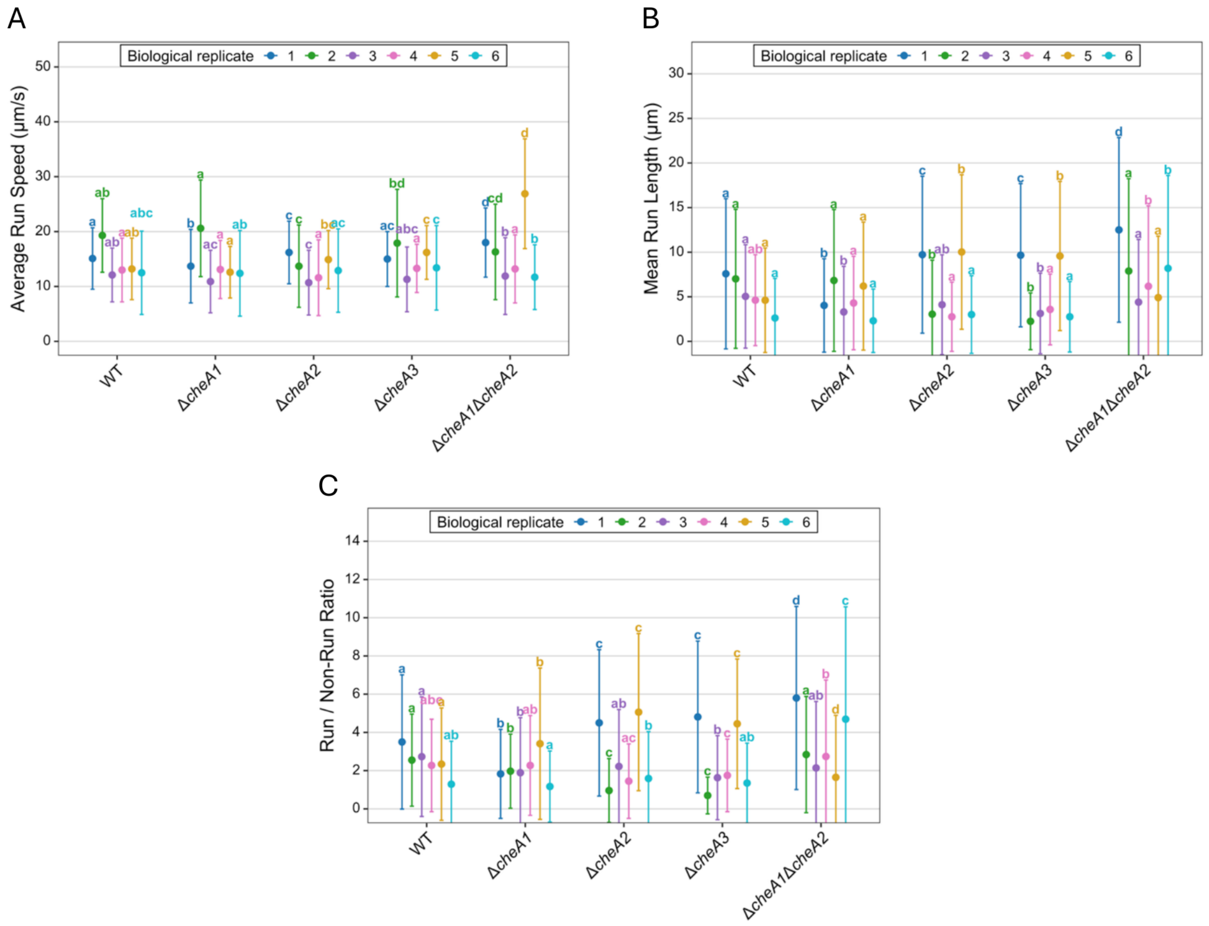


**Figure S3. Swimming motility in aqueous medium is not impacted by loss of *cheA* homologs.** **(A)** Dot-and-whisker plots of the average per-track run speed (µm/s), **(B)** mean per-track run length (µm), and **(C)** per-track run-to-non-run frame ratio for single cells of WT, Δ*cheA1*, Δ*cheA2*, Δ*cheA3*, and Δ*cheA1*Δ*cheA2*, with each strain represented by six replicates. Superscript letters represent statistically indistinguishable groups ($p > 0.05$). Numerical summary statistics for these distributions are provided in **Table 2**.

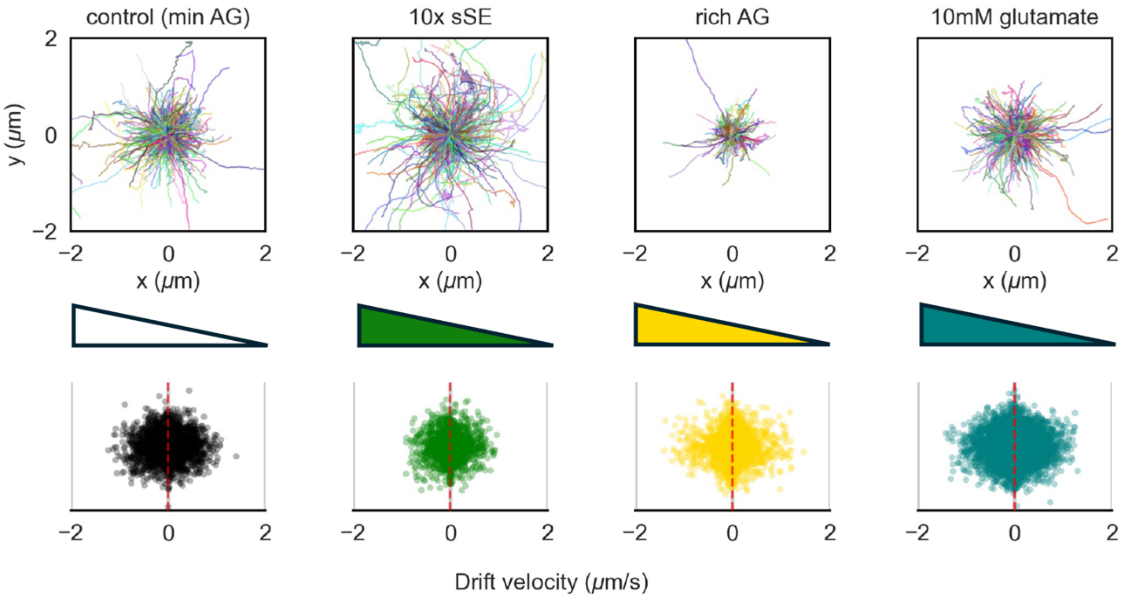


**Figure S4. Chemotaxis is not observed in microscopy assay conditions. (A)** Rose plots of single-cell swimming trajectories for WT cells in μ-Chemotaxis slides (ibidi) under four different gradient conditions: minimal AG medium on both sides (control, "homogeneous"); 10X sSE in the right chamber; rich AG medium in the right chamber; or 10 mM glutamate in the right chamber. For each condition, 2,000 randomly sampled tracks (after length and stuck-cell filtering) are shown, each translated to a common origin at (0,0); axes are in μm with a fixed range of ±2 μm. Triangular schematics below each panel indicate the orientation of the chemoattractant gradient. **(B)** Per-track drift velocity along the gradient axis ($\partial x/\partial t$ in μm/s) for cells shown in (A), plotted as jittered scatter. Each point represents one track; the red dashed vertical line indicates zero net drift (*i.e.*, equal swimming in the positive and negative x directions).

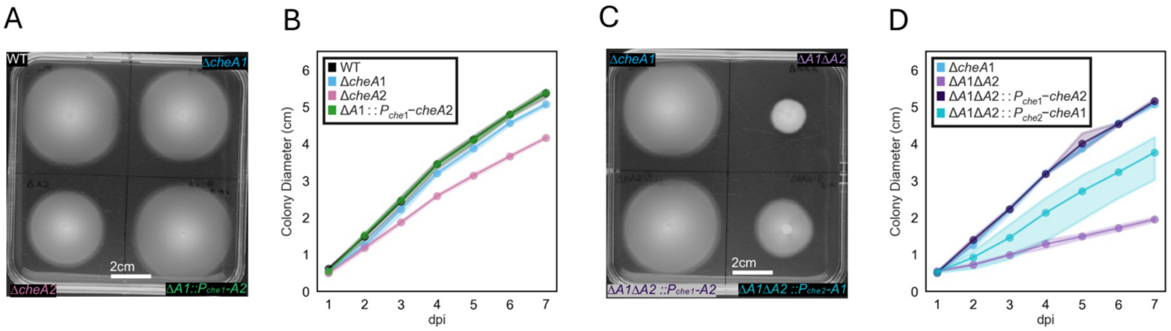


**Figure S5. Loss of che2 operon function, not loss of the *cheA2* coding sequence, is responsible for reduced expansion in different *cheA* genomic backgrounds. (A)** Representative images at 7 dpi of expansion halos for WT, Δ*cheA1*, Δ*cheA2*, and the swap strain Δ*cheA1*::P*che1*-*cheA2* (in which the native *cheA1* coding sequence is replaced by *cheA2* under control of the endogenous *che1* promoter and regulatory elements) on a single 12-cm square semisolid minimal AG plate. **(B)** Expansion-halo diameter over 7 days for the strains in (A); points are means across N=3 plates per strain, shaded bands indicate the range of observed values . **(C)** Representative images at 7 dpi of Δ*cheA1*Δ*cheA2* alongside two complementation strains generated in the Δ*cheA1*Δ*cheA2* double-deletion background: Δ*cheA1*Δ*cheA2*::P*che1*-*cheA2* (restores *cheA2* expression from the *che1* locus) and Δ*cheA1*Δ*cheA2*::P*che2*-*cheA1* (restores *cheA1* expression from the *che2* locus). **(D)** Expansion-halo diameter over 7 days for Δ*cheA1* (reference WT-like control), Δ*cheA1*Δ*cheA2*, Δ*cheA1*Δ*cheA2*::P*che1*-*cheA2*, and Δ*cheA1*Δ*cheA2*::P*che2*-*cheA1*. Points are means across N=3 plates per strain; shaded bands indicate the range of observed values. Δ*cheA1*Δ*cheA2*::P*che2*-*cheA1* (i.e., a functional *che2* operon expressing *cheA1*) restores Δ*cheA1*-like (≈WT) expansion, while Δ*cheA1*Δ*cheA2*::P*che1*-*cheA2* (a functional *che1* operon expressing *cheA2*) phenocopies the slow-expanding Δ*cheA1*Δ*cheA2* parent.
