# Supplementary material for: Characterization of chemotaxis in soybean symbiont Bradyrhizobium diazoefficiens
Source: Appl Environ Microbiol. 2026 Jun 24;92(7):e00928-26. doi: 10.1128/aem.00928-26 (PMC13390405; doi:10.1128/aem.00928-26)
Supplement: Supplemental legends — Descriptive legends for Videos S1 to S5. [file aem.00928-26-s0002.docx]

**SUPPLEMENTAL VIDEO LEGENDS**

**Video S1.** Representative swimming wild-type *B. diazoefficiens* cells. Cells stained with SYTO9, resuspended in motility medium, and imaged using confocal fluorescence microscopy at ~17 frames per second. Scale bar, 5 μm.

**Video S2.** Representative swimming Δ*cheA1* cells prepared as described for Video S1.

**Video S3.** Representative swimming Δ*cheA2* cells prepared as described for Video S1.

**Video S4.** Representative swimming Δ*cheA3* cells prepared as described for Video S1.

**Video S5.** Representative swimming Δ*cheA1*Δ*cheA2* prepared as described for Video S1.
