# Supplementary material for: Characterization of chemotaxis in soybean symbiont Bradyrhizobium diazoefficiens
Source: Appl Environ Microbiol. 2026 Jun 24;92(7):e00928-26. doi: 10.1128/aem.00928-26 (PMC13390405; doi:10.1128/aem.00928-26)
Supplement: Supplemental tables — Tables S1 to S4. [file aem.00928-26-s0003.docx]

| **Strain** | **Genotype** | **Parent strain** | **Construction** | **Source / reference** |
| --- | --- | --- | --- | --- |
| WT | *Bradyrhizobium diazoefficiens* USDA110 *spc*4 (wild type) | — (parental) | Spontaneous spectinomycin-resistant derivative of USDA110. | BacDive ID 10.13145/bacdive133983.20251217.10 |
| Δ*fla* (LP 6543) | USDA110 *str* Δ*lafA12*::Sm-Sp^R^ Δ*fliC1234*::Km^R^ | LP 6865 (USDA110 *str* Δ*lafA12*::Sm-Sp^R^) | Constructed in the laboratory of A. R. Lodeiro (UNLP, Argentina). | Althabegoiti et al., 2011 (27); Quelas et al., 2016 (30); Garrido-Sanz et al., 2019 (56). |
| Δ*cheA1* | USDA110 *spc*4 Δ*cheA1* (Δbll0393) | USDA110 *spc*4 | Marker-less deletion of *cheA1* by allelic exchange using suicide vector pk18sacB_delcheA1 (Table S4). | This study |
| Δ*cheA2* | USDA110 *spc*4 Δ*cheA2* (Δblr2192) | USDA110 *spc*4 | Marker-less deletion of *cheA2* by allelic exchange using suicide vector pk18sacB_delcheA2 (Table S4). | This study |
| Δ*cheA3* | USDA110 *spc*4 Δ*cheA3* (Δblr2343) | USDA110 *spc*4 | Marker-less deletion of *cheA3* by allelic exchange using suicide vector pk18sacB_delcheA3 (Table S4). | This study |
| Δ*cheA1*Δ*cheA2* | USDA110 *spc*4 Δ*cheA1* Δ*cheA2*  (Δbll0393 Δblr2192) | Δ*cheA1* | Sequential allelic exchange: deletion of *cheA2* in the Δ*cheA1* background using pk18sacB_delcheA2 (Table S4). | This study |
| Δ*cheA1*Δ*cheA3* | USDA110 *spc*4 Δ*cheA1* Δ*cheA3*  *(*Δbll0393 Δblr2343) | Δ*cheA1* | Sequential allelic exchange: deletion of *cheA3* in the Δ*cheA1* background using pk18sacB_delcheA3 (Table S4). | This study |
| Δ*cheA2*Δ*cheA3* | USDA110 *spc*4 Δ*cheA2* Δ*cheA3 (*Δblr2192 Δblr2343) | Δ*cheA2* | Sequential allelic exchange: deletion of *cheA3* in the Δ*cheA2* background using pk18sacB_delcheA3 (Table S4). | This study |
| Δ*cheA1*Δ*cheA2*Δ*cheA3* | USDA110 *spc*4 Δ*cheA1* Δ*cheA2* Δ*cheA3*  (Δbll0393 Δblr2192 Δblr2343) | Δ*cheA1*Δ*cheA2* | Sequential allelic exchange: deletion of *cheA3* in the Δ*cheA1*Δ*cheA2* double-deletion background using pk18sacB_delcheA3 (Table S4). | This study |
| Δ*cheA2*::P*che2*-*cheA1* | USDA110 *spc*4 Δ*cheA2*, with *cheA1* coding sequence integrated at the *che2* locus | Δ*cheA2* | Allelic exchange in the Δ*cheA2* background using pk18sacB_A1-in-che2 (Table S4) to replace the deleted *cheA2* ORF with the *cheA1* ORF at the *che2* locus. | This study |
| Δ*cheA1*::P*che1*-*cheA2* | USDA110 *spc*4 Δ*cheA1*, with *cheA2* coding sequence integrated at the *che1* locus | Δ*cheA1* | Allelic exchange in the Δ*cheA1* background using pk18sacB_A2-in-che1 (Table S4) to replace the deleted *cheA1* ORF with the *cheA2* ORF at the *che1* locus. | This study |
| Δ*cheA1*Δ*cheA2*::P*che1*-*cheA2* | USDA110 *spc*4 Δ*cheA1* Δ*cheA2*, with *cheA2* coding sequence integrated at the *che1* locus | Δ*cheA1*Δ*cheA2* | Allelic exchange in the Δ*cheA1*Δ*cheA2* double-deletion background using pk18sacB_A2-in-che1 (Table S4) to integrate the *cheA2* ORF at the *che1* locus. | This study |
| Δ*cheA1*Δ*cheA2*::P*che2*-*cheA1* | USDA110 *spc*4 Δ*cheA1* Δ*cheA2*, with *cheA1* coding sequence integrated at the *che2* locus | Δ*cheA1*Δ*cheA2* | Allelic exchange in the Δ*cheA1*Δ*cheA2* double-deletion background using pk18sacB_A1-in-che2 (Table S4) to integrate the *cheA1* ORF at the *che2* locus. | This study |
| *E. coli* S17-1 λ*pir* | *recA*, *thi*, *pro*, *hsdR*⁻*M*⁺ RP4-2-Tc::Mu-Km::Tn7 λ*pir* | conjugation donor strain | Used for conjugative transfer of pK18*mobsacB*-derived suicide vectors into *B. diazoefficiens*. | ATCC (47055) |

**Table S1.** Bacterial strains used in this study**.**

| **Primer Name** | **Gene Target** | **Sequence** |
| --- | --- | --- |
| sigA_qRT_F | sigA (bll7349) | AATGAAGTGTTGCCGTCCGA |
| sigA_qRT_R | sigA (bll7349) | TTGATGCCCATGTCCGAGAG |
| lafA_F | lafA/flgE (blr3700) | CTTCAGGTGGCATCGTCCAT |
| lafA_R | lafA/flgE (blr3700) | CGTGGTCAGACCGAGCTTAT |
| fliC_F | fliC (bll6855) | TGGATTGACGCAATACGCCT |
| fliC_R | fliC (bll6855) | CCGTTCGAGTAGGTGGCATC |
| cheA1_RTPCR_F | cheA1 (bll0393) | CCATGGAGAGCGTCACTGTT |
| cheA1_RTPCR_R | cheA1 (bll0393) | CTCGATGTTGAGGCGTTCCT |
| cheA2_RTPCR_F | cheA2 (blr2192) | CCATGGAGAGCGTCACTGTT |
| cheA2_RTPCR_R | cheA2 (blr2192) | CTCGATGTTGAGGCGTTCCT |
| cheA3_RTPCR_F | cheA3 (blr2343) | GCTGGCACCTGTATCTGGAA |
| cheA3_RTPCR_R | cheA3 (blr2343) | AGCTTGACGTCCCACTTCAG |

**Table S2.** Primers used for RT-qPCR in this study.

| **Replicate** | **Strain** | **Raw track n** | **Post-filtering track n** | **Percent retained** |
| --- | --- | --- | --- | --- |
| 1 | WT | 12473 | 1931 | 15.48 |
| 1 | *∆cheA1* | 13647 | 962 | 7.05 |
| 1 | *∆cheA2* | 4868 | 781 | 16.04 |
| 1 | *∆cheA3* | 4813 | 417 | 8.66 |
| 1 | *∆A1∆A2* | 14291 | 2017 | 14.11 |
| 2 | WT | 3083 | 1344 | 43.59 |
| 2 | *∆cheA1* | 1492 | 390 | 26.14 |
| 2 | *∆cheA2* | 793 | 225 | 28.37 |
| 2 | *∆cheA3* | 5225 | 775 | 14.83 |
| 2 | *∆A1∆A2* | 1283 | 181 | 14.11 |
| 3 | WT | 4519 | 359 | 7.94 |
| 3 | *∆cheA1* | 10431 | 1063 | 10.19 |
| 3 | *∆cheA2* | 10473 | 684 | 6.53 |
| 3 | *∆cheA3* | 7252 | 361 | 4.98 |
| 3 | *∆A1∆A2* | 21147 | 1246 | 5.89 |
| 4 | WT | 3025 | 239 | 7.9 |
| 4 | *∆cheA1* | 8284 | 757 | 9.14 |
| 4 | *∆cheA2* | 2648 | 83 | 3.13 |
| 4 | *∆cheA3* | 4309 | 527 | 12.23 |
| 4 | *∆A1∆A2* | 2424 | 260 | 10.73 |
| 5 | WT | 4597 | 535 | 11.64 |
| 5 | *∆cheA1* | 3442 | 325 | 9.44 |
| 5 | *∆cheA2* | 3458 | 411 | 11.89 |
| 5 | *∆cheA3* | 4297 | 785 | 18.27 |
| 5 | *∆A1∆A2* | 55197 | 13373 | 24.23 |
| 6 | WT | 21803 | 1507 | 6.91 |
| 6 | *∆cheA1* | 18489 | 2176 | 11.77 |
| 6 | *∆cheA2* | 12228 | 1594 | 13.04 |
| 6 | *∆cheA3* | 18857 | 1918 | 10.17 |
| 6 | *∆A1∆A2* | 21907 | 1132 | 5.17 |

**Table S3.** Summary of track filtering to remove non-motile cell tracks. For each replicate, for each strain, the table reports the number of individual cell tracks captured by TumbleScore with no filtering (“Raw track n”) and counts surviving the subsequent non-motile-cell filtering (“Post-filtering track n”).

| **pk18sacB_delcheA1** | | | For deletion of Δ*cheA1* (Δbll0393) | |
| --- | --- | --- | --- | --- |
| ATTCATGACGCCGCATGGCTGCAGATTTCGCGTCCGTCGTTTCCGTCCTCCTGGCTTCGCCAAAGACGTTCGCGACCGTCAGTTCTTCGATCCCAACCGGAGGAGAAAGGCGTGCCCCACGGTTCAATTATTGAATCGCCGAAACGGGAAGCTCAAACGGGGACAGAAAGTCTGCGCCATATTCTCGTTGTCGACGACGACCCGATGGTGTGCATGGCCATCGAGGTCTATCTTCAGCTAAACCATTTTCGGGTGACGATTGCCGAAGGAGGAGAGGTCGGACTGCGTGCTCTCGAACACGGACAGTTCGATCTGATGATCATCGACATCTTCATGCCGCACATGCGCGGCTTCGAATCGATCAGGGTGTTCCACGAGCGGGCGCCCACCATTCCGTTGATCGCGATGTCCGGCTACGCTTTCGCAAATCTGAATTCACCCGCTCCCGATTTCCTCCGGATGGCGCTCGAGCTCGGCGCGGCACGCTGTCTGCGCAAGCCGTTCACGCCGCACGCTCTGCTCGCCGCCGTCAACGATTGCTTCGCGGAGCACCGCTCCGACGACGTTGCCTCGGCCGCGCGCCTGGGTTAGCGCAGCGCAGGCCGTCGTAGCGGCGGGGTAAGCGTCTGTTTACCACAAACGCGGGCCTTGCGGATCCCGGCAGGGCACGCCGCGACGGTCGCGAGAGCGCTCCCGATCGGCTTCGATTTCACGTGGGCCGTGCATATCGTCCTTCACGAGCAGGACTTTCTTGCGCGCGTCGTCGCGCCGTTGTCGGCGCGTTTACCTAACGCTTCTTTTGGTGACAGTTCGGTGCCGGTTTTACCGAGAAGCGCTGCCGCATGTGATCGAACCACAAACTTCTCTTCAATATCCGTATTAGCACGGAGGATGAAATTAACGGGACCGTGAAAGGGGTTGTCTAACGATCCAGACGGGGACCTCCGTCGATGCCAAGCGTGGCTCAGGCGTCGAAGTCCAGCTCAGTAAGGCGTAGCTCGAGCATGATCCGGAAAAGTGTGAAGCGGTTTTCCGACGAGATCATGCTCAAAACGAAAAAGCGGGATCAGGAGATACGCTGATGGCCAACAAGACCCAGTCCACCGAAGGCGCCATGGTCGAATACGTCACCGCGATGATCGGCGGCCAGCTGTTCGGCCTGCCGATCTCCCGCGTCCAGGACGTGTTCATGCCCGAGCGCGTCACCCGCGTTCCGCTGTCCTCCCGCGAGATCGCCGGCGTCCTCAACCTGCGCGGCCGCATCGTCACCGTGGTCGACATGCGCGCCCGCCTCGGCCTGCCGAGGCCCGAGGACGGCAAGGTGCCGATGGCGGTCGGTGTCGACCTGCGCGGCGAATCCTATGGCCTGCTGATCGACCAGATCGGCGAGGTGCTGCGCCTGCCCGAGGACGGCAAGGAAGAAAACCCTGTCAACCTCGACCCCCGCATGGCCAAGCTCGCCGGCGGTGTCCACCGCCTCGACGGCCAGCTCATGGTCGTCCTCGACGTCGATCGCGTCCTCGAGCTCGAAACCAAAGTGCAAATGGCTGCGTGAACCAACGAAACATCGAAGCCGGAGAACCAAAATGAAGACCTGTTTGGTGGTCGATGATTCCAGCGTCGTGCGCAAGATCGCGCGCCGCATCCTGGAAGGCCTGGAATTCCAGGTCACCGAAGCCGAGGACGGCTCGAAGGCGCTCGAGATCTGCCAGCGGCAACTGCCCGATGCGGTGTTGCTCGACTGGAACATGCCCGTGATGGACGGCTTCGAGTTCATGGGCCACATGCGCCGCCTGCCCGGCGGCGACCAGCCGAAGGTGGTGTTCTGCACCACCGAGAACAACGTGGCCCATATCGCCCAGGCGCTCAGCGGCGGCGCCAACGAATACATCATGAAGCCCTTCGACAAAGACATTATCGCCGACAAGTTCGCTGAAGTCGGCTTGATCCCGGTCGGACAAGCCATGGTCTGAGTGTGTCTGGCCCGAGTGTTCCGGCCA | | | | |
| **pk18sacB_delcheA2** | | | For deletion of Δ*cheA2* (Δblr2192) | |
| TTCGCCCCTGGCTTTATGGCCGCCCCGAACCGATCGCAAGCTAGCTGCCGCAGGCTTGCGAATGTTAGAACCCGCAGCATCCGTCAAGCATGCGTGATTCGCGGCGTCCAGCGCCGTGCAATTCCAGGAACCCTCCAGGACCCCATTATATGTCTGACGCCTCGTCGCCCGCGACCGCTACTGCTCCCGATATGCTCGAACTCGCCGCGCTGCTGTGCTCGCGGGTCTGCCACGATCTCATCAGCCCCGTCGGCGCCATCGTCAACGGGCTCGAAGTGCTCGATGACGATCCCAAGCCCGAGGACCGCGAGTTCGCGCTCGACCTGATTCGCAAGAGCGCCAAGACGGCCTCCGCCCGCCTCCAGTTCTGCCGTCTCGCCTTCGGCGCCGCCGGCTCCTCCGGCGCCCAGATCGACCTCGGCGATGCCCAGACCATGGCGCGCGGCCACATCGAGGACGGCAAGTGCACGATCACGTGGAATCTGCCGCGGCTGCTGCTGCCGAAGAATCGCGTCAAGCTGCTGCTCAACATGCTGGTCGTGGCCCAGCACACGATCCCGCGCGGCGGTACGCTGACGATCGATCCGGTCGGCGAGGGCGAGACGATGAGCTTTCGCATCACCGCGACCGGGCACAACGCGCGCCTGCCGCAGAACATCGCCGAGCTCCTGAGCGGCGAGCGCGGGCCCGCTGCGGATGCGCACGCGATCCAGCCTTATTATACGCGGCTGCTCGCGCAGGCCTGCGGGCTCACCGTGACGCTGGCACCGGAAGGCGCAGCCATCATCGTTACCGCTTCGTAAACGCGCGCATCGCTCTCAAGACGTTAATCGAATCTTTACGAGGCGCTTCGGCTTGTCCGGAGCGCCTTATCTCTTTGTTGGTTCCGTTCTTTTCCACATACTCAACCATTATTAAACGCTTTGCGGTGAAGCTGGCCTCATTCCGAAATGGCGCATCACGCCTCGTGCGCGCCCTCCCTGTATGAAGGCCTGTTTTCGAGCATGATCCGGAAAAGTGTGAAGCGGTTTTCCGACGAGATCATGCTCAAAACGAAAAAGCGGGATCAGGAGATACGCTGATGGCCAACAAGACCCAGTCCACCGAAGGCGCCATGGTCGAATACGTCACCGCGATGATCGGCGGCCAGCTGTTCGGCCTGCCGATCTCCCGCGTCCAGGACGTGTTCATGCCCGAGCGCGTCACCCGCGTTCCGCTGTCCTCCCGCGAGATCGCCGGCGTCCTCAACCTGCGCGGCCGCATCGTCACCGTGGTCGACATGCGCGCCCGCCTCGGCCTGCCGAGGCCCGAAGACGGCAAGGTGCCGATGGCGGTCGGTGTCGATCTGCGCGGCGAATCCTATGGCCTGCTGATCGACCAGATCGGCGAGGTGCTGCGCCTGCCCGAGGACGGCAAGGAAGAAAACCCCGTCAACCTCGACCCCCGCATGGCCAAGCTCGCCGGCGGTGTCCACCGCCTCGACGGCCAGCTCATGGTCGTCCTCGACGTCGATCGCGTCCTCGAGCTCGCGCCCGAGATGATGGCGGCCTGATACGGCGGCACTGCCGCCGACGGACTTGCAATTGGAAGTGTCCCCGCGAAGGGGACAGGAAGCAGAGGTTCACATGCGCACTTGTCTCGTCGTTGATGATTCCAGCGTCATCCGCAAGGTCGCGCGCCGCATCCTGGAGGGCCTCGACTTCCAGATCCTCGAAGCCGAGGACGGTGAGAAGGCGCTGGAGGCCTGCAAGCGCGGCTTGCCCGACGCGGTGCTGCTGGACTGGAACATGCCTGTCATGGACGGCTACGAGTTCCTCGGCCATCTCCGGCGCATGCCCGGCGGCGACCAGCCCAAGGTGGTGTTCTGCACCACCGAGAACGACGTTGCGCACATCGCGCGTGCGCTTCATGCCGGCGCCAACGAGTACATCATGAAGCCCTTCGACAAGGACATCGTGACGGCGAAGTTCCAGGAAGTCGGCCTGATCTGAGCGACCGTC | | | | |
| **pk18sacB_delcheA3** | | | | For deletion of Δ*cheA3* (Δblr2343) |
| CCTCGCATGCGATTTCAGCAGGCTATGCACCGTTGCATGCAATTGCGCGAAGTCCTCGGGAATGTGACCCGTAGTTGTACGGACCTCTCCGTTAGGGGTTTGCTCACTTGATTCGCGTCAAACGTGTATCGGAATTTTGCAAGGGCGGGGGCAGCGATGGCGCTCAATCCGACGGAGCCGAAGTGGTCGTTGCGGTTACCCGCCGATTGCAGCATCGCGGCCATTCGCAGCGTCTATGACCTCATTCGCGAGGCGTTCGGCCGCGAGCAGCGGCTCGAGATCGATTGTTCGCGCGTCGACAAGGCCGACGTGACCTCGATCCAGCTTCTGCTGTCGACCGCCAAGACGGGCGAGGCCCAGGGCCGCCCGGTGGTGCTCACCGCATTCTCCCAATCTCTGCGCAACACCCTTCGCCGCGCCGGCTTCGCCAGCGAGGCGATGATCGAACAGCACTTCCCGCAAAAGAAAGATGGCACCTGATGGCCACGATTCTGACCGTCGACGATTCCCCCAGCATTCGGCAAATGATCAAGGTCGTGCTCGAGCCGGCCGGTCATAACGTGATCGAGGCCGGTGACGGTGCGCAAGGGCTCGCCAAGGCCCAGGCCGGCAAGCTCGACCTCGTGATCACCGACCTCAATATGCCGGTCATGAACGGGCTGGAGCTGATCCGGGCGCTGCGCAAGCTGCCGAGCGCGGTCGGCATGCCGATCGTGTTCCTGACCACCGAATCCAACGACACGGTGAAGCAGGAGGCCAAGAGCGCCGGCGCCACGGGCTGGATCACCAAGCCCTTCAAGCCGGAGCAGTTGCTCGCCGTCGTCGGCAAGCTGGTGCGCGCATGAGTGCAATGGACCCGACCGAGGTCTTTCGTCAGGAAGCCAGTGAGCTCTTCGAAGTTCTTGAAGGAGCCCTGCTCGATCTCGGCCTGCGTCCCGATGACCGCGAACTCGTCGATTCCGCCTTCCGCGCCCTGCATACGATCAAGGGCTCGGGCGCCTGAACGAGACTTTGGCCGGCGAGCATCAGGCCGACGCGATGCAGGTCGTGATGATCGGCCTCGGCGAGGAGAAGTTCGCGCTCGACGCGGGCCTCGTGCGCGAGATCATCGATCCCGTGCCGGTGACCAAGGTCGCCGGCGCACGGGCTTTCGTTCCCAGCGTGATCAACGTACGCGGCAACGTCATTCCGCTCGCGGACCTGCGCATCCGCTTCGGTATGCCGCAGCTCGACAACTCGGCCGACACGCGCATCGTCGTCATCGAGCTGGAGCTCGACGGCGAGCCCGTCCTGGTCGGCGTCACCGCCGACAAGGTCTATGAGGTCACGGAGATCTCGCAGACCGACGTGCAGCAGACGCCGCGCGTCGGCATGCACTGGAAGCCGGAGTTCATTCGCTTCATCGCCAAATGGCGTGAAGAGTTCGTCATCGTTCCCAACATGGAACGCATTCTGAATTGAAATGAGTCCCAGGGGTGGGACTTGAGGTTTAGGGGCTGGAAATGAGATTTACGGTCAAAGCGAAGCTGGCCAGCGCATTCGGCCTGGTCATTGTCCTGTCCATGGTCGCCGGCGGTGTCGCGTACATGAAGCTCGGCGACATGATGGCCACTGCTGACAGCATGGTCCTGCGCGCGACCCGGATGGAGAAGGCGACGCAAATCGAAAAGGATATCCTGCTTCAGCTGCGGGCGGAGAAGAATTCAATTCTCGGAGCGGAAGCCCAGGCCGAACAGTTTGCCGCCGATGCCGCCAAGATTCGTGACCAGGCGCTGAAGACCAAGGACGAGGTCTACGCGTTGGCGAGCGAAGCCGGCAGGAAGCTGCTCGACAGCTTCGCTGTGACATACGCCAAGATGAATGTGTATCAGGAAGAGACGTTCAGACTGGCCAAGACCGACAAGGCAAAGGCGACCGAACGCTCCATGGGCGACGGTCGCAAGGTCGTCGCGGACGCGATGGAAGCCATGAGCGCCTATGTCGGCAACACGAAAAA | | | | |
| **pk18sacB_A1-in-che2** | | For insertion of the *cheA1* (Δbll0393) coding sequence in the che2 operon | | |
| TGCCGCAAGCACTCAGGGCGCAAGGGCTGCTAAAGGAAGCGGAACACGTAGAAAGCCAGTCCGCAGAAACGGTGCTGACCCCGGATGAATGTCAGCTACTGGGCTATCTGGACAAGGGAAAACGCAAGCGCAAAGAGAAAGCAGGTAGCTTGCAGTGGGCTTACATGGCGATAGCTAGACTGGGCGGTTTTATGGACAGCAAGCGAACCGGAATTGCCAGCTGGGGCGCCCTCTGGTAAGGTTGGGAAGCCCTGCAAAGTAAACTGGATGGCTTTCTTGCCGCCAAGGATCTGATGGCGCAGGGGATCAAGATCTGATCAAGAGACAGGATGAGGATCGTTTCGCATGATTGAACAAGATGGATTGCACGCAGGTTCTCCGGCCGCTTGGGTGGAGAGGCTATTCGGCTATGACTGGGCACAACAGACAATCGGCTGCTCTGATGCCGCCGTGTTCCGGCTGTCAGCGCAGGGGCGCCCGGTTCTTTTTGTCAAGACCGACCTGTCCGGTGCCCTGAATGAACTCCAAGACGAGGCAGCGCGGCTATCGTGGCTGGCCACGACGGGCGTTCCTTGCGCAGCTGTGCTCGACGTTGTCACTGAAGCGGGAAGGGACTGGCTGCTATTGGGCGAAGTGCCGGGGCAGGATCTCCTGTCATCTCACCTTGCTCCTGCCGAGAAAGTATCCATCATGGCTGATGCAATGCGGCGGCTGCATACGCTTGATCCGGCTACCTGCCCATTCGACCACCAAGCGAAACATCGCATCGAGCGAGCACGTACTCGGATGGAAGCCGGTCTTGTCGATCAGGATGATCTGGACGAAGAGCATCAGGGGCTCGCGCCAGCCGAACTGTTCGCCAGGCTCAAGGCGCGGATGCCCGACGGCGAGGATCTCGTCGTGACCCATGGCGATGCCTGCTTGCCGAATATCATGGTGGAAAATGGCCGCTTTTCTGGATTCATCGACTGTGGCCGGCTGGGTGTGGCGGACCGCTATCAGGACATAGCGTTGGCTACCCGTGATATTGCTGAAGAGCTTGGCGGCGAATGGGCTGACCGCTTCCTCGTGCTTTACGGTATCGCCGCTCCCGATTCGCAGCGCATCGCCTTCTATCGCCTTCTTGACGAGTTCTTCTGAGCGGGACTCTGGGGTTCGCTAGAGGATCGATCCTTTTTAACCCATCACATATACCTGCCGTTCACTATTATTTAGTGAAATGAGATATTATGATATTTTCTGAATTGTGATTAAAAAGGCAACTTTATGCCCATGCAACAGAAACTATAAAAAATACAGAGAATGAAAAGAAACAGATAGATTTTTTAGTTCTTTAGGCCCGTAGTCTGCAAATCCTTTTATGATTTTCTATCAAACAAAAGAGGAAAATAGACCAGTTGCAATCCAAACGAGAGTCTAATAGAATGAGGTCGAAAAGTAAATCGCGCGGGTTTGTTACTGATAAAGCAGGCAAGACCTAAAATGTGTAAAGGGCAAAGTGTATACTTTGGCGTCACCCCTTACATATTTTAGGTCTTTTTTTATTGTGCGTAACTAACTTGCCATCTTCAAACAGGAGGGCTGGAAGAAGCAGACCGCTAACACAGTACATAAAAAAGGAGACATGAACGATGAACATCAAAAAGTTTGCAAAACAAGCAACAGTATTAACCTTTACTACCGCACTGCTGGCAGGAGGCGCAACTCAAGCGTTTGCGAAAGAAACGAACCAAAAGCCATATAAGGAAACATACGGCATTTCCCATATTACACGCCATGATATGCTGCAAATCCCTGAACAGCAAAAAAATGAAAAATATCAAGTTTCTGAATTTGATTCGTCCACAATTAAAAATATCTCTTCTGCAAAAGGCCTGGACGTTTGGGACAGCTGGCCATTACAAAACGCTGACGGCACTGTCGCAAACTATCACGGCTACCACATCGTCTTTGCATTAGCCGGAGATCCTAAAAATGCGGATGACACATCGATTTACATGTTCTATCAAAAAGTCGGCGAAACTTCTATTGACAGCTGGAAAAACGCTGGCCGCGTCTTTAAAGACAGCGACAAATTCGATGCAAATGATTCTATCCTAAAAGACCAAACACAAGAATGGTCAGGTTCAGCCACATTTACATCTGACGGAAAAATCCGTTTATTCTACACTGATTTCTCCGGTAAACATTACGGCAAACAAACACTGACAACTGCACAAGTTAACGTATCAGCATCAGACAGCTCTTTGAACATCAACGGTGTAGAGGATTATAAATCAATCTTTGACGGTGACGGAAAAACGTATCAAAATGTACAGCAGTTCATCGATGAAGGCAACTACAGCTCAGGCGACAACCATACGCTGAGAGATCCTCACTACGTAGAAGATAAAGGCCACAAATACTTAGTATTTGAAGCAAACACTGGAACTGAAGATGGCTACCAAGGCGAAGAATCTTTATTTAACAAAGCATACTATGGCAAAAGCACATCATTCTTCCGTCAAGAAAGTCAAAAACTTCTGCAAAGCGATAAAAAACGCACGGCTGAGTTAGCAAACGGCGCTCTCGGTATGATTGAGCTAAACGATGATTACACACTGAAAAAAGTGATGAAACCGCTGATTGCATCTAACACAGTAACAGATGAAATTGAACGCGCGAACGTCTTTAAAATGAACGGCAAATGGTACCTGTTCACTGACTCCCGCGGATCAAAAATGACGATTGACGGCATTACGTCTAACGATATTTACATGCTTGGTTATGTTTCTAATTCTTTAACTGGCCCATACAAGCCGCTGAACAAAACTGGCCTTGTGTTAAAAATGGATCTTGATCCTAACGATGTAACCTTTACTTACTCACACTTCGCTGTACCTCAAGCGAAAGGAAACAATGTCGTGATTACAAGCTATATGACAAACAGAGGATTCTACGCAGACAAACAATCAACGTTTGCGCCGAGCTTCCTGCTGAACATCAAAGGCAAGAAAACATCTGTTGTCAAAGACAGCATCCTTGAACAAGGACAATTAACAGTTAACAAATAAAAACGCAAAAGAAAATGCCGATGGGTACCGAGCGAAATGACCGACCAAGCGACGCCCAACCTGCCATCACGAGATTTCGATTCCACCGCCGCCTTCTATGAAAGGTTGGGCTTCGGAATCGTTTTCCGGGACGCCCTCGCGGACGTGCTCATAGTCCACGACGCCCGTGATTTTGTAGCCCTGGCCGACGGCCAGCAGGTAGGCCGACAGGCTCATGCCGGCCGCCGCCGCCTTTTCCTCAATCGCTCTTCGTTCGTCTGGAAGGCAGTACACCTTGATAGGTGGGCTGCCCTTCCTGGTTGGCTTGGTTTCATCAGCCATCCGCTTGCCCTCATCTGTTACGCCGGCGGTAGCCGGCCAGCCTCGCAGAGCAGGATTCCCGTTGAGCACCGCCAGGTGCGAATAAGGGACAGTGAAGAAGGAACACCCGCTCGCGGGTGGGCCTACTTCACCTATCCTGCCCGGCTGACGCCGTTGGATACACCAAGGAAAGTCTACACGAACCCTTTGGCAAAATCCTGTATATCGTGCGAAAAAGGATGGATATACCGAAAAAATCGCTATAATGACCCCGAAGCAGGGTTATGCAGCGGAAAAGCGCTGCTTCCCTGCTGTTTTGTGGAATATCTACCGACTGGAAACAGGCAAATGCAGGAAATTACTGAACTGAGGGGACAGGCGAGAGACGATGCCAAAGAGCTCCTGAAAATCTCGATAACTCAAAAAATACGCCCGGTAGTGATCTTATTTCATTATGGTGAAAGTTGGAACCTCTTACGTGCCGATCAACGTCTCATTTTCGCCAAAAGTTGGCCCAGGGCTTCCCGGTATCAACAGGGACACCAGGATTTATTTATTCTGCGAAGTGATCTTCCGTCACAGGTATTTATTCGGCGCAAAGTGCGTCGGGTGATGCTGCCAACTTACTGATTTAGTGTATGATGGTGTTTTTGAGGTGCTCCAGTGGCTTCTGTTTCTATCAGCTCCTGAAAATCTCGATAACTCAAAAAATACGCCCGGTAGTGATCTTATTTCATTATGGTGAAAGTTGGAACCTCTTACGTGCCGATCAACGTCTCATTTTCGCCAAAAGTTGGCCCAGGGCTTCCCGGTATCAACAGGGACACCAGGATTTATTTATTCTGCGAAGTGATCTTCCGTCACAGGTATTTATTCGGCGCAAAGTGCGTCGGGTGATGCTGCCAACTTACTGATTTAGTGTATGATGGTGTTTTTGAGGTGCTCCAGTGGCTTCTGTTTCTATCAGGGCTGGATGATCCTCCAGCGCGGGGATCTCATGCTGGAGTTCTTCGCCCACCCCAAAAGGATCTAGGTGAAGATCCTTTTTGATAATCTCATGACCAAAATCCCTTAACGTGAGTTTTCGTTCCACTGAGCGTCAGACCCCGTAGAAAAGATCAAAGGATCTTCTTGAGATCCTTTTTTTCTGCGCGTAATCTGCTGCTTGCAAACAAAAAAACCACCGCTACCAGCGGTGGTTTGTTTGCCGGATCAAGAGCTACCAACTCTTTTTCCGAAGGTAACTGGCTTCAGCAGAGCGCAGATACCAAATACTGTTCTTCTAGTGTAGCCGTAGTTAGGCCACCACTTCAAGAACTCTGTAGCACCGCCTACATACCTCGCTCTGCTAATCCTGTTACCAGTGGCTGCTGCCAGTGGCGATAAGTCGTGTCTTACCGGGTTGGACTCAAGACGATAGTTACCGGATAAGGCGCAGCGGTCGGGCTGAACGGGGGGTTCGTGCACACAGCCCAGCTTGGAGCGAACGACCTACACCGAACTGAGATACCTACAGCGTGAGCTATGAGAAAGCGCCACGCTTCCCGAAGGGAGAAAGGCGGACAGGTATCCGGTAAGCGGCAGGGTCGGAACAGGAGAGCGCACGAGGGAGCTTCCAGGGGGAAACGCCTGGTATCTTTATAGTCCTGTCGGGTTTCGCCACCTCTGACTTGAGCGTCGATTTTTGTGATGCTCGTCAGGGGGGCGGAGCCTATGGAAAAACGCCAGCAACGCGGCCTTTTTACGGTTCCTGGCCTTTTGCTGGCCTTTTGCTCACATGTTCTTTCCTGCGTTATCCCCTGATTCTGTGGATAACCGTATTACCGCCTTTGAGTGAGCTGATACCGCTCGCCGCAGCCGAACGACCGAGCGCAGCGAGTCAGTGAGCGAGGAAGCGGAAGAGCGCCCAATACGCAAACCGCCTCTCCCCGCGCGTTGGCCGATTCATTAATGCAGCTGGCACGACAGGTTTCCCGACTGGAAAGCGGGCAGTGAGCGCAACGCAATTAATGTGAGTTAGCTCACTCATTAGGCACCCCAGGCTTTACACTTTATGCTTCCGGCTCGTATGTTGTGTGGAATTGTGAGCGGATAACAATTTCACACAGGAAACAGCTATGACATGATTACGAATTCTTCGCCCCTGGCTTTATGGCCGCCCCGAACCGATCGCAAGCTAGCTGCCGCAGGCTTGCGAATGTTAGAACCCGCAGCATCCGTCAAGCATGCGTGATTCGCGGCGTCCAGCGCCGTGCAATTCCAGGAACCCTCCAGGACCCCATTATATGTCTGACGCCTCGTCGCCCGCGACCGCTACTGCTCCCGATATGCTCGAACTCGCCGCGCTGCTGTGCTCGCGGGTCTGCCACGATCTCATCAGCCCCGTCGGCGCCATCGTCAACGGGCTCGAAGTGCTCGATGACGATCCCAAGCCCGAGGACCGCGAGTTCGCGCTCGACCTGATTCGCAAGAGCGCCAAGACGGCCTCCGCCCGCCTCCAGTTCTGCCGTCTCGCCTTCGGCGCCGCCGGCTCCTCCGGCGCCCAGATCGACCTCGGCGATGCCCAGACCATGGCGCGCGGCCACATCGAGGACGGCAAGTGCACGATCACGTGGAATCTGCCGCGGCTGCTGCTGCCGAAGAATCGCGTCAAGCTGCTGCTCAACATGCTGGTCGTGGCCCAGCACACGATCCCGCGCGGCGGTACGCTGACGATCGATCCGGTCGGCGAGGGCGAGACGATGAGCTTTCGCATCACCGCGACCGGGCACAACGCGCGCCTGCCGCAGAACATCGCCGAGCTCCTGAGCGGCGAGCGCGGGCCCGCTGCGGATGCGCACGCGATCCAGCCTTATTATACGCGGCTGCTCGCGCAGGCCTGCGGGCTCACCGTGACGCTGGCACCGGAAGGCGCAGCCATCATCGTTACCGCTTCGTAAACGCGCGCATCGCTCTCAAGACGTTAATCGAATCTTTACGAGGCGCTTCGGCTTGTCCGGAGCGCCTTATCTCTTTGTTGGTTCCGTTCTTTTCCACATACTCAACCATTATTAAACGCTTTGCGGTGAAGCTGGCCTCATTCCGAAATGGCGCATCACGCCTCGTGCGCGCCCTCCCTGTATGAAGGCCTGTTTTCATGGATGATCTGTTGCGGGAGTTCTTGACGGAGACCAGCGAGAGCCTGGACACCGTCGACAATCAGCTGGTGAAGTTCGAGCAGGAGCCGAACAACGCCAAGATCCTGGATAACATCTTCCGCCTGGTCCACACCATCAAGGGGACGTGCGGCTTCCTCGGCTTGCCGCGACTGGAAGCGCTGGCGCATGCCGGCGAGACGCTGATGGGCAAGTTCCGCGACGGCATGCCGGTGACGGGGCAGGCGGTGACGGTGATCCTGTCCTCGATCGACCGCATCAAGGAGATCCTCGCCGGCCTCGAGGCGACCGAAGCCGAGCCGGAGGGCACCGACCGCGATCTCATCGACAAGCTGGAAGCGATGGTCGAGCAGGGCATGGCGGCGATGTCAGCGTCGGCTTCGCCGATCGCGTCAGGCTCGGCGCAGCCGATGCCGGCGGCTGGCAGCGCCGCTGCTGTTGCTGACGCGCCGCCGCTGGTGCCGGAAGCGCCGGCCGCCGCTGCGCCGGCCAAGGACATGACCACGGGTTCGCTGATCGACCAGACCCTGGAGCGCCCGCTGCGCCCGGGTGAAGTGTCGCTCGACGAGCTCGAGCGCGCCTTCCGCGAAACCGCGATCGAAGCCCCAATCCCCGCGCCAATCGTCAAGGCCGAGGTCAAGGCTGAGCCCGCGCCGGCTCCGGCCCCTGTTGCCAAAGAGGCTGCCAAGGAAGCTGCGAAGCCTGCCGCCAAGGAGAAGGCCGCGCCGAAGAAGTCGATGGCCGACGAGGGCGCCTCCGAGGGCGACCGCATCGCCAACCAGTCGATCCGCGTCAACGTGGATACGCTGGAGCATCTGATGACCATGGTCTCCGAGCTGGTCTTGACCCGCAACCAGCTGCTGGAGATCTCCCGCCGCAATGAGGACACCGAGTTCAAGGTGCCGTTGCAGCGCCTCTCCAACGTCACCGCCGAGCTGCAGGAAGGCGTCATGAAGACGCGCATGCAGCCGATCGGCAATGCCTGGCAGAAGCTGCCCCGCATCGTCCGCGACCTGTCGAGCGAACTCGGCAAGCAGATCGAGCTGGAGATGCACGGCGCCGACACCGAGCTCGACCGCCAGGTGCTCGACCTGATCAAGGACCCGCTCACCCACATGGTGCGCAACTCCGCCGACCATGGCCTGGAGACCCCCGCCGAGCGGCTCGCGGCCGGCAAGGGCGAGCAGGGCACCATCCGCCTCTCCGCCTATCACGAGGGCGGCCACATCATCATCTGCATCGCCGACAACGGCCGCGGCCTCAACACCGAGAGGATCAAGGCCAAGGCGATCTCCTCGGGTCTCGTCACCGAGGCCGAGCTCGAGAAGATGAGCGAAGCCCAGATCCACAAGTTCATCTTCGCGCCGGGCTTCTCGACCGCGGCCGCCATCACCTCGGTGTCGGGCCGCGGCGTCGGCATGGACGTGGTGCGCACCAATATCGACCAGATCGGCGGCACCATCGACATCAAGTCGGTGGCCGGCGAGGGCTCGAGCGTCACCATCAAGATCCCGCTGACCTTGGCCATCGTCTCCGCGCTGATCGTGGAAGCCGCCGGCGACCGCTTTGCGATCCCGCAGCTCTCCGTCGTCGAGCTGGTCCGGGCCCGCGCCAACAGCGAGCACCGCATCGAGCGCATCAAGGACACCGCCGTCCTGCGCCTGCGCAACAAGCTCTTGCCGCTGATCCATTTGAAGAAGCTGCTCAAGATCGACGACGGCGCGGCCAGCGATCCCGAGAACGGTTTTATCGTGGTGACGCAGGTCGGCAGCCAGACCTTCGGCATCGTCGTCGACGGCGTCTTCCACACCGAAGAAATCGTGGTCAAGCCGATGTCGACGAAGCTGCGTCACATCGACATGTTCTCCGGCAACACCATTCTGGGCGATGGCGCCGTCATCATGATCATCGACCCCAACGGCATTGCCAAGGCGCTGGGTGCTGCCGGCTCCTCGGCCCATGACATGGGCGACGAGAACGGGGCGCATCACATCGGAAGTGGCGAGCAGACCACTTCGCTGCTGGTGTTCCGCGCCGGCTCGTCCCAGCCCAAGGCGGTCCCGCTCGGGCTCGTCACGCGCCTGGAGGAGCTGCCCGCCGACAAGATCGAGTTCAGCAACGGCCGCTACATGGTGCAGTACCGCGAGCAGCTGATGCCGCTCGTCGCCATGGAGAGCGTCACTGTTGCGAGCCAAGGCGCCCAGCCGATCCTGGTGTTCGCCGACGACGGCCGCTCCATGGGCCTCGTCGTCGACGAGATCATCGACATCGTCGAGGAACGCCTCAACATCGAGGTCGGCGGCTCCAGCCAGGGCATCCTGGGCTCGGCCGTGATCAAGGGGCAGGCCACCGAGGTGATCGACGTCGGCCACTTCCTGCCGATGGCGTTCGCCGACTGGTTCACCCGCAAGGAGATGAAGCCGTCGATGCATTCGCAGTCGGTGCTGCTGGTCGACGATTCCGCGTTCTTCCGCAACATGCTGGCGCCGGTGCTGAAAGCCGCCGGCTACCGCGTCCGCACCGCGCCGACCGCGCAGGAGGGCCTGGCCGCGCTGCGCGCCCAGAGCTTCGACGTGGTCCTGACCGACATCGAGATGCCCGACATGAACGGGTTCGAGTTCGCCGAGGTGATCCGCTCCGACAACAATCTCGGCGCGATGCCGATCATCGGCCTGTCCGCGCTGGTGTCGCCGGCGGCGATCGAGCGCGGCCGTCAGGCCGGCTTCCACGACTATGTCGCCAAGTTCGACCGTCCCGGTCTGATCGCGGCGCTGAAGGAGCAGACCGCGGGCGCCGCCGGCGCCTCCGAGCTGAGCCGGGCAGCGGCGTAAGAGCATGATCCGGAAAAGTGTGAAGCGGTTTTCCGACGAGATCATGCTCAAAACGAAAAAGCGGGATCAGGAGATACGCTGATGGCCAACAAGACCCAGTCCACCGAAGGCGCCATGGTCGAATACGTCACCGCGATGATCGGCGGCCAGCTGTTCGGCCTGCCGATCTCCCGCGTCCAGGACGTGTTCATGCCCGAGCGCGTCACCCGCGTTCCGCTGTCCTCCCGCGAGATCGCCGGCGTCCTCAACCTGCGCGGCCGCATCGTCACCGTGGTCGACATGCGCGCCCGCCTCGGCCTGCCGAGGCCCGAAGACGGCAAGGTGCCGATGGCGGTCGGTGTCGATCTGCGCGGCGAATCCTATGGCCTGCTGATCGACCAGATCGGCGAGGTGCTGCGCCTGCCCGAGGACGGCAAGGAAGAAAACCCCGTCAACCTCGACCCCCGCATGGCCAAGCTCGCCGGCGGTGTCCACCGCCTCGACGGCCAGCTCATGGTCGTCCTCGACGTCGATCGCGTCCTCGAGCTCGCGCCCGAGATGATGGCGGCCTGATACGGCGGCACTGCCGCCGACGGACTTGCAATTGGAAGTGTCCCCGCGAAGGGGACAGGAAGCAGAGGTTCACATGCGCACTTGTCTCGTCGTTGATGATTCCAGCGTCATCCGCAAGGTCGCGCGCCGCATCCTGGAGGGCCTCGACTTCCAGATCCTCGAAGCCGAGGACGGTGAGAAGGCGCTGGAGGCCTGCAAGCGCGGCTTGCCCGACGCGGTGCTGCTGGACTGGAACATGCCTGTCATGGACGGCTACGAGTTCCTCGGCCATCTCCGGCGCATGCCCGGCGGCGACCAGCCCAAGGTGGTGTTCTGCACCACCGAGAACGACGTTGCGCACATCGCGCGTGCGCTTCATGCCGGCGCCAACGAGTACATCATGAAGCCCTTCGACAAGGACATCGTGACGGCGAAGTTCCAGGAAGTCGGCCTGATCTGAGCGACCGTCAAGCTTGGCACTGGCCGTCGTTTTACAACGTCGTGACTGGGAAAACCCTGGCGTTACCCAACTTAATCGCCTTGCAGCACATCCCCCTTTCGCCAGCTGGCGTAATAGCGAAGAGGCCCGCACCGATCGCCCTTCCCAACAGTTGCGCAGCCTGAATGGCGAATGGCGATAAGCTAGCTTCACGC | | | | |
| **pk18sacB_A2-in-che1** | For insertion of *cheA2* (Δblr2192) coding sequence in the che1 operon | | | |
| GCGTGAAGCTAGCTGGCCGGAACACTCGGGCCAGACACACTCAGACCATGGCTTGTCCGACCGGGATCAAGCCGACTTCAGCGAACTTGTCGGCGATAATGTCTTTGTCGAAGGGCTTCATGATGTATTCGTTGGCGCCGCCGCTGAGCGCCTGGGCGATATGGGCCACGTTGTTCTCGGTGGTGCAGAACACCACCTTCGGCTGGTCGCCGCCGGGCAGGCGGCGCATGTGGCCCATGAACTCGAAGCCGTCCATCACGGGCATGTTCCAGTCGAGCAACACCGCATCGGGCAGTTGCCGCTGGCAGATCTCGAGCGCCTTCGAGCCGTCCTCGGCTTCGGTGACCTGGAATTCCAGGCCTTCCAGGATGCGGCGCGCGATCTTGCGCACGACGCTGGAATCATCGACCACCAAACAGGTCTTCATTTTGGTTCTCCGGCTTCGATGTTTCGTTGGTTCACGCAGCCATTTGCACTTTGGTTTCGAGCTCGAGGACGCGATCGACGTCGAGGACGACCATGAGCTGGCCGTCGAGGCGGTGGACACCGCCGGCGAGCTTGGCCATGCGGGGGTCGAGGTTGACAGGGTTTTCTTCCTTGCCGTCCTCGGGCAGGCGCAGCACCTCGCCGATCTGGTCGATCAGCAGGCCATAGGATTCGCCGCGCAGGTCGACACCGACCGCCATCGGCACCTTGCCGTCCTCGGGCCTCGGCAGGCCGAGGCGGGCGCGCATGTCGACCACGGTGACGATGCGGCCGCGCAGGTTGAGGACGCCGGCGATCTCGCGGGAGGACAGCGGAACGCGGGTGACGCGCTCGGGCATGAACACGTCCTGGACGCGGGAGATCGGCAGGCCGAACAGCTGGCCGCCGATCATCGCGGTGACGTATTCGACCATGGCGCCTTCGGTGGACTGGGTCTTGTTGGCCATCAGCGTATCTCCTGATCCCGCTTTTTCGTTTTGAGCATGATCTCGTCGGAAAACCGCTTCACACTTTTCCGGATCATGCTCTTACGCCGCTGCCCGGCTCAGCTCGGAGGCGCCGGCGGCGCCCGCGGTCTGCTCCTTCAGCGCCGCGATCAGACCGGGACGGTCGAACTTGGCGACATAGTCGTGGAAGCCGGCCTGACGGCCGCGCTCGATCGCCGCCGGCGACACCAGCGCGGACAGGCCGATGATCGGCATCGCGCCGAGATTGTTGTCGGAGCGGATCACCTCGGCGAACTCGAACCCGTTCATGTCGGGCATCTCGATGTCGGTCAGGACCACGTCGAAGCTCTGGGCGCGCAGCGCGGCCAGGCCCTCCTGCGCGGTCGGCGCGGTGCGGACGCGGTAGCCGGCCGCTTTCAGGACCGGCGCCAGCATGTTGCGGAAGAACGCGGAGTCGTCGACCAGCAGCACCGACTGCGAGTGCATCGACGGCTTCATCTCCTTGCGGGTGAACCAGTCGGCGAACGCCATCGGCAGGAAGTGGCCGACGTCAATCACCTCGGTGGCCTGCCCCTTGATCACGGCCGAGCCCAGGATGCCCTGGCTGGAGCCGCCGACCTCGATGTTGAGGCGTTCCTCGACGATGTCGATGATCTCGTCGACGACGAGGCCCATGGAGCGGCCGTCGTCGGCGAACACCAGGATCGGCTGGGCGCCTTGGCTCGCAACAGTGACGCTCTCCATGGCGACGAGCGGCATCAGCTGCTCGCGGTACTGCACCATGTAGCGGCCGTTGCTGAACTCGATCTTGTCGGCGGGGAGCTCTTCCAGGCGCGTAACGAGCCCGAGCGGGACCGCCTTGGGCTGGGACGAGCCGGCGCGGAACACCAGCAGCGAAGTGGTCTGCTCGCCCGATCCGATGTGATGCGCCCCGTTCTCGTCGCCCATGTCATGGGCCGAGGAGCCGGCGGCACCCAGCGCCTTGGCAATGCCGTTGGGGTCGATGATCATGATGACGGCGCCATCGCCCAGGATGGTGTTGCCGGAGAACATGTCGATGTGACGCAGTTTTGTCGACATCGGCTTGACCACGATTTCTTCGGTATGGAAGACGCCGTCGACGACGATGCCGAAGGTCTGGCTGCCGACCTGCGTCACCACGATAAAACCGTTCTCGGGATCGCTGGCCGCGCCGTCGTCGATCTTGAGCAGCTTCTTCAAATGGATCAGCGGCAAGAGCTTGTTGCGCAGGCGCAGGACGGCGGTGTCCTTGATGCGCTCGATGCGGTGCTCGCTGTTGGCGCGGGCCCGGACCAGCTCGACGACGGAGAGCTGCGGGATCGCGAAGCGGTCGCCGGCGGCTTCCACGATCAGCGCGGAGACGATGGCCAAGGTCAGCGGGATCTTGATGGTGACGCTCGAGCCCTCGCCGGCCACCGACTTGATGTCGATGGTGCCGCCGATCTGGTCGATATTGGTGCGCACCACGTCCATGCCGACGCCGCGGCCCGACACCGAGGTGATGGCGGCCGCGGTCGAGAAGCCCGGTGCGAAGATGAACTTGTGGATCTGGGCTTCGCTCATCTTCTCGAGCTCAGCCTCGGTGACGAGACCCGAGGAGATCGCCTTGGCCTTGATCCTCTCGGTGTTGAGGCCGCGGCCGTTGTCGGCGATGCAGATGATGATGTGGCCGCCCTCGTGATAGGCGGAGAGGCGGATGGTGCCCTGCTCGCCCTTGCCGGCCGCGAGCCGCTCGGCGGGGGTCTCCAGGCCATGGTCGGCGGAGTTGCGCACCATGTGGGTGAGCGGGTCCTTGATCAGGTCGAGCACCTGGCGGTCGAGCTCGGTGTCGGCGCCGTGCATCTCCAGCTCGATCTGCTTGCCGAGTTCGCTCGACAGGTCGCGGACGATGCGGGGCAGCTTCTGCCAGGCATTGCCGATCGGCTGCATGCGCGTCTTCATGACGCCTTCCTGCAGCTCGGCGGTGACGTTGGAGAGGCGCTGCAACGGCACCTTGAACTCGGTGTCCTCGTTGCGGCGGGAGATCTCCAGCAGCTGGTTGCGGGTCAAGACCAGCTCGGAGACCATGGTCATCAGATGCTCCAGCGTATCCACGTTGACGCGGATCGACTGGTTGGCGATGCGGTCGCCCTCGGAGGCGCCCTCGTCGGCCATCGACTTCTTCGGCGCGGCCTTCTCCTTGGCGGCAGGCTTCGCAGCTTCCTTGGCAGCCTCTTTGGCAACAGGGGCCGGAGCCGGCGCGGGCTCAGCCTTGACCTCGGCCTTGGCAACGGGTGCGGGGATTGGGGCTTCGATCGCGGTCTCGCGGAAGGCGCGCTCGAGCTCGTCCAGCGACACCTCGCCCGGGCGCAGCGGGCGCTCCAGGGTCTGATCGATCAGTGAACCCGTGGTCATGTCCTTGGCCGGCGCAGCGGCGGCCGGAGCTTCCGGCACCAGCGGCGGGGCGTCAGCAACAGCAGTAGCGCTGCCAGCCGCCGGCATCGGCTGCGCCGAGCCTGACGCGATCGGCGAAGCCGACGCTGACATCGCCGCCATGCCCTGCTCGACCATCGCTTCCAGCTTGTCGATGAGATCGCGGTCGGTGCCCTCCGGCTCGGCTTCGGTCGCCTCGAGGCCGGCGAGGATCTCCTTGATGCGGTCGATCGAGGACAGGATCACCGTCACCGCCTGCCCCGTCACCGGCATGCCGTCGCGGAACTTGCCCATCAGCGTCTCGCCGGCATGCGCCAGCGCTTCCAGTCGCGGCAGGCCGAGGAAGCCGCACGTCCCCTTGATGGTGTGGACCAGGCGGAAGATGTTATCCAGGATCTTGGCGTTGTTCGGCTCCTGCTCGAACTTCACCAGCTGATTGTCGACGGTGTCCAGGCTCTCGCTGGTCTCCGTCAAGAACTCCCGCAACAGATCATCCATGAGCTACGCCTTACTGAGCTGGACTTCGACGCCTGAGCCACGCTTGGCATCGACGGAGGTCCCCGTCTGGATCGTTAGACAACCCCTTTCACGGTCCCGTTAATTTCATCCTCCGTGCTAATACGGATATTGAAGAGAAGTTTGTGGTTCGATCACATGCGGCAGCGCTTCTCGGTAAAACCGGCACCGAACTGTCACCAAAAGAAGCGTTAGGTAAACGCGCCGACAACGGCGCGACGACGCGCGCAAGAAAGTCCTGCTCGTGAAGGACGATATGCACGGCCCACGTGAAATCGAAGCCGATCGGGAGCGCTCTCGCGACCGTCGCGGCGTGCCCTGCCGGGATCCGCAAGGCCCGCGTTTGTGGTAAACAGACGCTTACCCCGCCGCTACGACGGCCTGCGCTGCGCTAACCCAGGCGCGCGGCCGAGGCAACGTCGTCGGAGCGGTGCTCCGCGAAGCAATCGTTGACGGCGGCGAGCAGAGCGTGCGGCGTGAACGGCTTGCGCAGACAGCGTGCCGCGCCGAGCTCGAGCGCCATCCGGAGGAAATCGGGAGCGGGTGAATTCAGATTTGCGAAAGCGTAGCCGGACATCGCGATCAACGGAATGGTGGGCGCCCGCTCGTGGAACACCCTGATCGATTCGAAGCCGCGCATGTGCGGCATGAAGATGTCGATGATCATCAGATCGAACTGTCCGTGTTCGAGAGCACGCAGTCCGACCTCTCCTCCTTCGGCAATCGTCACCCGAAAATGGTTTAGCTGAAGATAGACCTCGATGGCCATGCACACCATCGGGTCGTCGTCGACAACGAGAATATGGCGCAGACTTTCTGTCCCCGTTTGAGCTTCCCGTTTCGGCGATTCAATAATTGAACCGTGGGGCACGCCTTTCTCCTCCGGTTGGGATCGAAGAACTGACGGTCGCGAACGTCTTTGGCGAAGCCAGGAGGACGGAAACGACGGACGCGAAATCTGCAGCCATGCGGCGTCATGAATTCTAGAGGATCCCCGGGTACCGAGCTCGAATTCGTAATCATGTCATAGCTGTTTCCTGTGTGAAATTGTTATCCGCTCACAATTCCACACAACATACGAGCCGGAAGCATAAAGTGTAAAGCCTGGGGTGCCTAATGAGTGAGCTAACTCACATTAATTGCGTTGCGCTCACTGCCCGCTTTCCAGTCGGGAAACCTGTCGTGCCAGCTGCATTAATGAATCGGCCAACGCGCGGGGAGAGGCGGTTTGCGTATTGGGCGCTCTTCCGCTTCCTCGCTCACTGACTCGCTGCGCTCGGTCGTTCGGCTGCGGCGAGCGGTATCAGCTCACTCAAAGGCGGTAATACGGTTATCCACAGAATCAGGGGATAACGCAGGAAAGAACATGTGAGCAAAAGGCCAGCAAAAGGCCAGGAACCGTAAAAAGGCCGCGTTGCTGGCGTTTTTCCATAGGCTCCGCCCCCCTGACGAGCATCACAAAAATCGACGCTCAAGTCAGAGGTGGCGAAACCCGACAGGACTATAAAGATACCAGGCGTTTCCCCCTGGAAGCTCCCTCGTGCGCTCTCCTGTTCCGACCCTGCCGCTTACCGGATACCTGTCCGCCTTTCTCCCTTCGGGAAGCGTGGCGCTTTCTCATAGCTCACGCTGTAGGTATCTCAGTTCGGTGTAGGTCGTTCGCTCCAAGCTGGGCTGTGTGCACGAACCCCCCGTTCAGCCCGACCGCTGCGCCTTATCCGGTAACTATCGTCTTGAGTCCAACCCGGTAAGACACGACTTATCGCCACTGGCAGCAGCCACTGGTAACAGGATTAGCAGAGCGAGGTATGTAGGCGGTGCTACAGAGTTCTTGAAGTGGTGGCCTAACTACGGCTACACTAGAAGAACAGTATTTGGTATCTGCGCTCTGCTGAAGCCAGTTACCTTCGGAAAAAGAGTTGGTAGCTCTTGATCCGGCAAACAAACCACCGCTGGTAGCGGTGGTTTTTTTGTTTGCAAGCAGCAGATTACGCGCAGAAAAAAAGGATCTCAAGAAGATCCTTTGATCTTTTCTACGGGGTCTGACGCTCAGTGGAACGAAAACTCACGTTAAGGGATTTTGGTCATGAGATTATCAAAAAGGATCTTCACCTAGATCCTTTTGGGGTGGGCGAAGAACTCCAGCATGAGATCCCCGCGCTGGAGGATCATCCAGCCCTGATAGAAACAGAAGCCACTGGAGCACCTCAAAAACACCATCATACACTAAATCAGTAAGTTGGCAGCATCACCCGACGCACTTTGCGCCGAATAAATACCTGTGACGGAAGATCACTTCGCAGAATAAATAAATCCTGGTGTCCCTGTTGATACCGGGAAGCCCTGGGCCAACTTTTGGCGAAAATGAGACGTTGATCGGCACGTAAGAGGTTCCAACTTTCACCATAATGAAATAAGATCACTACCGGGCGTATTTTTTGAGTTATCGAGATTTTCAGGAGCTGATAGAAACAGAAGCCACTGGAGCACCTCAAAAACACCATCATACACTAAATCAGTAAGTTGGCAGCATCACCCGACGCACTTTGCGCCGAATAAATACCTGTGACGGAAGATCACTTCGCAGAATAAATAAATCCTGGTGTCCCTGTTGATACCGGGAAGCCCTGGGCCAACTTTTGGCGAAAATGAGACGTTGATCGGCACGTAAGAGGTTCCAACTTTCACCATAATGAAATAAGATCACTACCGGGCGTATTTTTTGAGTTATCGAGATTTTCAGGAGCTCTTTGGCATCGTCTCTCGCCTGTCCCCTCAGTTCAGTAATTTCCTGCATTTGCCTGTTTCCAGTCGGTAGATATTCCACAAAACAGCAGGGAAGCAGCGCTTTTCCGCTGCATAACCCTGCTTCGGGGTCATTATAGCGATTTTTTCGGTATATCCATCCTTTTTCGCACGATATACAGGATTTTGCCAAAGGGTTCGTGTAGACTTTCCTTGGTGTATCCAACGGCGTCAGCCGGGCAGGATAGGTGAAGTAGGCCCACCCGCGAGCGGGTGTTCCTTCTTCACTGTCCCTTATTCGCACCTGGCGGTGCTCAACGGGAATCCTGCTCTGCGAGGCTGGCCGGCTACCGCCGGCGTAACAGATGAGGGCAAGCGGATGGCTGATGAAACCAAGCCAACCAGGAAGGGCAGCCCACCTATCAAGGTGTACTGCCTTCCAGACGAACGAAGAGCGATTGAGGAAAAGGCGGCGGCGGCCGGCATGAGCCTGTCGGCCTACCTGCTGGCCGTCGGCCAGGGCTACAAAATCACGGGCGTCGTGGACTATGAGCACGTCCGCGAGGGCGTCCCGGAAAACGATTCCGAAGCCCAACCTTTCATAGAAGGCGGCGGTGGAATCGAAATCTCGTGATGGCAGGTTGGGCGTCGCTTGGTCGGTCATTTCGCTCGGTACCCATCGGCATTTTCTTTTGCGTTTTTATTTGTTAACTGTTAATTGTCCTTGTTCAAGGATGCTGTCTTTGACAACAGATGTTTTCTTGCCTTTGATGTTCAGCAGGAAGCTCGGCGCAAACGTTGATTGTTTGTCTGCGTAGAATCCTCTGTTTGTCATATAGCTTGTAATCACGACATTGTTTCCTTTCGCTTGAGGTACAGCGAAGTGTGAGTAAGTAAAGGTTACATCGTTAGGATCAAGATCCATTTTTAACACAAGGCCAGTTTTGTTCAGCGGCTTGTATGGGCCAGTTAAAGAATTAGAAACATAACCAAGCATGTAAATATCGTTAGACGTAATGCCGTCAATCGTCATTTTTGATCCGCGGGAGTCAGTGAACAGGTACCATTTGCCGTTCATTTTAAAGACGTTCGCGCGTTCAATTTCATCTGTTACTGTGTTAGATGCAATCAGCGGTTTCATCACTTTTTTCAGTGTGTAATCATCGTTTAGCTCAATCATACCGAGAGCGCCGTTTGCTAACTCAGCCGTGCGTTTTTTATCGCTTTGCAGAAGTTTTTGACTTTCTTGACGGAAGAATGATGTGCTTTTGCCATAGTATGCTTTGTTAAATAAAGATTCTTCGCCTTGGTAGCCATCTTCAGTTCCAGTGTTTGCTTCAAATACTAAGTATTTGTGGCCTTTATCTTCTACGTAGTGAGGATCTCTCAGCGTATGGTTGTCGCCTGAGCTGTAGTTGCCTTCATCGATGAACTGCTGTACATTTTGATACGTTTTTCCGTCACCGTCAAAGATTGATTTATAATCCTCTACACCGTTGATGTTCAAAGAGCTGTCTGATGCTGATACGTTAACTTGTGCAGTTGTCAGTGTTTGTTTGCCGTAATGTTTACCGGAGAAATCAGTGTAGAATAAACGGATTTTTCCGTCAGATGTAAATGTGGCTGAACCTGACCATTCTTGTGTTTGGTCTTTTAGGATAGAATCATTTGCATCGAATTTGTCGCTGTCTTTAAAGACGCGGCCAGCGTTTTTCCAGCTGTCAATAGAAGTTTCGCCGACTTTTTGATAGAACATGTAAATCGATGTGTCATCCGCATTTTTAGGATCTCCGGCTAATGCAAAGACGATGTGGTAGCCGTGATAGTTTGCGACAGTGCCGTCAGCGTTTTGTAATGGCCAGCTGTCCCAAACGTCCAGGCCTTTTGCAGAAGAGATATTTTTAATTGTGGACGAATCAAATTCAGAAACTTGATATTTTTCATTTTTTTGCTGTTCAGGGATTTGCAGCATATCATGGCGTGTAATATGGGAAATGCCGTATGTTTCCTTATATGGCTTTTGGTTCGTTTCTTTCGCAAACGCTTGAGTTGCGCCTCCTGCCAGCAGTGCGGTAGTAAAGGTTAATACTGTTGCTTGTTTTGCAAACTTTTTGATGTTCATCGTTCATGTCTCCTTTTTTATGTACTGTGTTAGCGGTCTGCTTCTTCCAGCCCTCCTGTTTGAAGATGGCAAGTTAGTTACGCACAATAAAAAAAGACCTAAAATATGTAAGGGGTGACGCCAAAGTATACACTTTGCCCTTTACACATTTTAGGTCTTGCCTGCTTTATCAGTAACAAACCCGCGCGATTTACTTTTCGACCTCATTCTATTAGACTCTCGTTTGGATTGCAACTGGTCTATTTTCCTCTTTTGTTTGATAGAAAATCATAAAAGGATTTGCAGACTACGGGCCTAAAGAACTAAAAAATCTATCTGTTTCTTTTCATTCTCTGTATTTTTTATAGTTTCTGTTGCATGGGCATAAAGTTGCCTTTTTAATCACAATTCAGAAAATATCATAATATCTCATTTCACTAAATAATAGTGAACGGCAGGTATATGTGATGGGTTAAAAAGGATCGATCCTCTAGCGAACCCCAGAGTCCCGCTCAGAAGAACTCGTCAAGAAGGCGATAGAAGGCGATGCGCTGCGAATCGGGAGCGGCGATACCGTAAAGCACGAGGAAGCGGTCAGCCCATTCGCCGCCAAGCTCTTCAGCAATATCACGGGTAGCCAACGCTATGTCCTGATAGCGGTCCGCCACACCCAGCCGGCCACAGTCGATGAATCCAGAAAAGCGGCCATTTTCCACCATGATATTCGGCAAGCAGGCATCGCCATGGGTCACGACGAGATCCTCGCCGTCGGGCATCCGCGCCTTGAGCCTGGCGAACAGTTCGGCTGGCGCGAGCCCCTGATGCTCTTCGTCCAGATCATCCTGATCGACAAGACCGGCTTCCATCCGAGTACGTGCTCGCTCGATGCGATGTTTCGCTTGGTGGTCGAATGGGCAGGTAGCCGGATCAAGCGTATGCAGCCGCCGCATTGCATCAGCCATGATGGATACTTTCTCGGCAGGAGCAAGGTGAGATGACAGGAGATCCTGCCCCGGCACTTCGCCCAATAGCAGCCAGTCCCTTCCCGCTTCAGTGACAACGTCGAGCACAGCTGCGCAAGGAACGCCCGTCGTGGCCAGCCACGATAGCCGCGCTGCCTCGTCTTGGAGTTCATTCAGGGCACCGGACAGGTCGGTCTTGACAAAAAGAACCGGGCGCCCCTGCGCTGACAGCCGGAACACGGCGGCATCAGAGCAGCCGATTGTCTGTTGTGCCCAGTCATAGCCGAATAGCCTCTCCACCCAAGCGGCCGGAGAACCTGCGTGCAATCCATCTTGTTCAATCATGCGAAACGATCCTCATCCTGTCTCTTGATCAGATCTTGATCCCCTGCGCCATCAGATCCTTGGCGGCAAGAAAGCCATCCAGTTTACTTTGCAGGGCTTCCCAACCTTACCAGAGGGCGCCCCAGCTGGCAATTCCGGTTCGCTTGCTGTCCATAAAACCGCCCAGTCTAGCTATCGCCATGTAAGCCCACTGCAAGCTACCTGCTTTCTCTTTGCGCTTGCGTTTTCCCTTGTCCAGATAGCCCAGTAGCTGACATTCATCCGGGGTCAGCACCGTTTCTGCGGACTGGCTTTCTACGTGTTCCGCTTCCTTTAGCAGCCCTTGCGCCCTGAGTGCTTGCGGCA | | | | |

**Table S4.** Sequences of vectors used for gene deletion.

**REFERENCE**

56. Garrido-Sanz D, Redondo-Nieto M, Mongiardini E, Blanco-Romero E, Durán D, Quelas JI, Martin M, Rivilla R, Lodeiro AR, Althabegoiti MJ. 2019. Phylogenomic analyses of Bradyrhizobium reveal uneven distribution of the lateral and subpolar flagellar systems, which extends to rhizobiales. Microorganisms 7:50. https://doi.org/10.3390/microorganisms7020050
